# Regio‐ and Diastereoselective C–C Silylation of Cyclopropyl Acetates Harnessing Fluorinated Poly(pyridyl)Borate Rhodium Catalysts

**DOI:** 10.1002/chem.202503217

**Published:** 2026-01-24

**Authors:** Vo Quang Huy Phan, Suman Das Adhikary, Junha Jeon, H. V. Rasika Dias

**Affiliations:** ^1^ Department of Chemistry and Biochemistry The University of Texas at Arlington Arlington Texas USA

**Keywords:** alkene ligands, C─C silylation, fluorinated ligands, homogeneous catalysis, rhodium

## Abstract

Rhodium complexes supported by fluorinated tris‐ and bis‐(pyridyl)borates effectively catalyze redox‐neutral C─C silylation of cyclopropyl acetates, producing the umpolung, silicon‐containing heterocyclic products, dioxasilolanes, in good yields with high regio‐ and diastereoselectivity. The enhanced performance arises from the weakly donating ability of the fluorinated ligands, along with the considerable steric bulk of the poly(pyridyl)borates, which together govern both reactivity and selectivity. Additionally, rhodium(I)‐cyclooctadiene and rhodium(I)‐norbornadiene complexes supported by poly(pyridyl)borate ligands have been isolated and characterized. X‐ray crystal structures show that in these square planar complexes, poly(pyridyl)borate ligands bind to rhodium in a κ^2^‐mode using two‐pyridyl arms, while the cyclic dienes coordinate in an *η* ^4^‐fashion.

## Graphical abstract

Fluorinated poly(pyridyl)borates have been utilized to stabilize rhodium‐diene complexes and as supporting ligands in rhodium‐catalyzed redox‐neutral C–C silylation of cyclopropyl acetates, producing silicon‐containing heterocyclic products, dioxasilolanes.

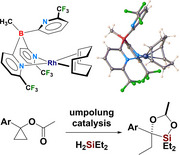

## 1 Introduction

**FIGURE 1:**
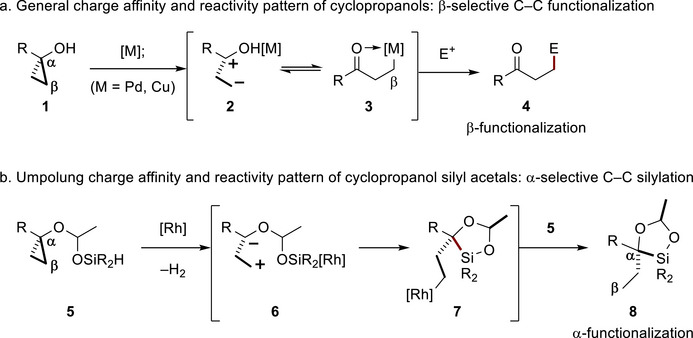
Catalytic redox‐neutral C─C silylation of cyclopropanol derivatives.

Catalytic carbon‐carbon (C─C) bond activation chemistry continues to revolutionize the quick and efficient assembly of complex molecular structures with wide‐ranging impacts in chemistry, biology, and medicine [1, 2, 3, 4, 5, 6, 7, 8, 9, 10, 11, 12, 13, 14]. Among the established protocols, C─C functionalization of cyclopropanols **1** [15, 16, 17, 18, 19] has emerged as a powerful strategy to access β‐functionalized ketones **4** via metallohomoenolate **3** [20, 21, 22, 23, 24, 25, 26]. This transformation is enabled by the formal charge affinity pattern in metalated cyclopropanols **2**, where the negative charge is located at the β‐position, facilitating regioselective functionalization (Figure 1a) [1, 2, 3, 4, 5, 6, 7, 8, 9, 10, 11, 12, 13, 14, 21, 27, 28, 29, 30, 31, 32, 33]. In our previous work, we disclosed redox‐neutral, catalytic α‐selective silylation of cyclopropanol silyl acetals **5**, resulting in the formation of dioxasilolanes **8** with high regio‐ and chemoselectivity (Figure 1b) [34]. This umpolung reactivity (i.e., polarity inverse) observed in rhoda silyl acetals (**6** to **7**) [35, 36, 37] was achieved by the π‐acidic rhodium catalyst supported by a highly fluorinated tris(pyrazolyl)borate ligand, [HB(3,5‐(CF_3_)_2_Pz)_3_]^−^ (**9**) [38, 39].

**FIGURE 2:**
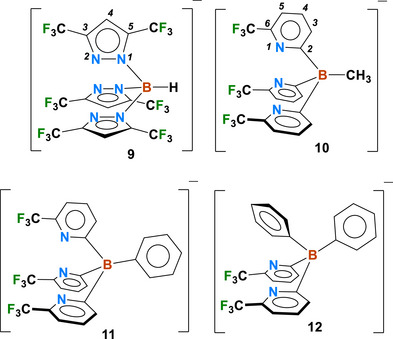
Fluorinated poly(pyrazolyl)borates and poly(pyridyl)borates, [HB(3,5‐(CF_3_)_2_Pz)_3_]^−^ (**9**), [MeB(6‐(CF_3_)Py)_3_]^−^ (**10**), [PhB(6‐(CF_3_)Py)_3_]^−^ (**11**), and [Ph_2_B(6‐(CF_3_)Py)_2_]^−^ (**12**), and the pyrazolyl (Pz) and pyridyl (Py) atom numbering scheme in these systems.

Unlike pyrazole‐based poly(pyrazolyl)borates [40, 41] such as **9**, pyridine‐based poly(pyridyl)borates [42] are a less explored category within the scorpionate family [40, 41]. The scarcity of ligand variations beyond those based on the parent 2‐pyridylborate is perhaps one of the reasons [42, 43]. Nevertheless, poly(2‐pyridyl)borates are finding increasing utility as metal ion chelators, as they offer different features relative to the pyrazolyl counterparts such as donor properties (pyridyl vs. pyrazolyl), backbone stabilities (attributable to less polar B─C linkages vs. B─N), ring‐substitution possibilities, and steric profiles (due to the involvement of six‐membered pyridyl donor arms instead of the five‐membered pyrazolyl moieties) [42]. Recently, we reported the isolation of fluorinated tris(2‐pyridyl)borate ligands such as [MeB(6‐(CF_3_)Py)_3_]^−^ (**10**, Py = pyridyl, Figure 2), [PhB(6‐(CF_3_)Py)_3_]^−^ (**11**), and bis(2‐pyridyl)borate ligands like [Ph_2_B(6‐(CF_3_)Py)_2_]^−^ (**12**), and demonstrated their utility in coinage metal chemistry [44, 45, 46, 47]. In the current study, we build on this work by examining how tris‐ and bis‐(pyridyl)borate ligands **10–12** influence the regio‐ and diastereoselective Rh‐catalyzed C─C silylation of cyclopropyl acetates. We have also synthesized and completely characterized four rhodium(I) complexes with olefinic ligands cod and nbd (cod = 1,5‐cyclooctadiene, nbd = norbornadiene) supported by **10–12**. It is noteworthy that a search of Cambridge Structural Database [48] returned over 200 hits for structurally characterized poly(pyrazolyl)borates, while a similar search involving poly(pyridyl)borate rhodium complexes produced only two hits, [Me_2_B(Py)_2_]Rh(CO)_2_ and [Me_2_B(Py)_2_]Rh(cod) [49].

## 2 Results and Discussion

The rhodium(I) complexes [MeB(6‐(CF_3_)Py)_3_]Rh(cod) (**13**), [PhB(6‐(CF_3_)Py)_3_]Rh(cod) (**14**), and [Ph_2_B(6‐(CF_3_)Py)_2_]Rh(cod) (**15**) were prepared by metathesis reactions using a 1:1 mixture of their respective potassium salts, [MeB(6‐(CF_3_)Py)_3_]K, [PhB(6‐(CF_3_)Py)_3_]K, and [Ph_2_B(6‐(CF_3_)Py)_2_]K, with [Rh(cod)Cl]_2_ in anhydrous dichloromethane at room temperature overnight. They were isolated as crystalline yellow‐orange solids in 72%, 61%, and 73% yields, respectively. Compound [MeB(6‐(CF_3_)Py)_3_]Rh(nbd) (**16**) was synthesized using [MeB(6‐(CF_3_)Py)_3_]K and [Rh(nbd)Cl]_2_ following an analogous method. These rhodium‐olefin compounds are soluble in common organic solvents such as CHCl_3_, CH_2_Cl_2_, C_6_H_6_, and THF, and are sparingly soluble in hexane.

**FIGURE 3:**
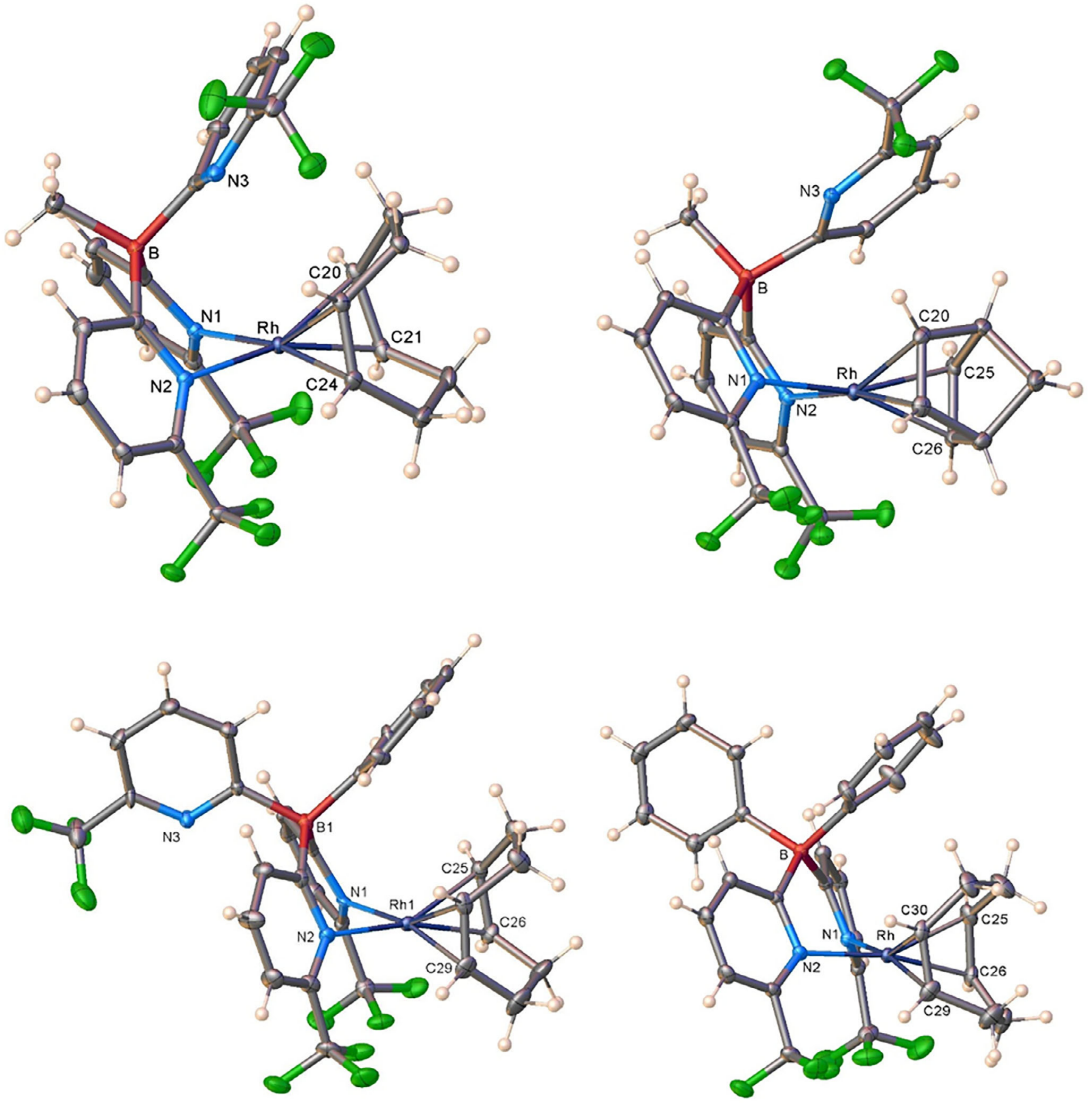
Molecular structures of [MeB(6‐(CF_3_)Py)_3_]Rh(cod) (**13,** top left), [MeB(6‐(CF_3_)Py)_3_]Rh(nbd) (**16,** top right), [PhB(6‐(CF_3_)Py)_3_]Rh(cod) (**14,** bottom left), and [Ph_2_B(6‐(CF_3_)Py)_2_]Rh(cod) (**15**, bottom right).

These molecules were characterized by X‐ray crystallography and NMR spectroscopy. Single crystal X‐ray analyses of **13–16** reveal that they are four‐coordinate square‐planar rhodium complexes with a six‐membered, boat‐shaped B(CN)_2_Rh metalacyclic core (Figure 3). The cyclooctadiene and norbornadiene ligands in **13** and **16**, respectively, exhibit *η* ^4^ coordination through two alkene groups on each ligand to rhodium. Similar *η* ^4^ coordination of cod observed in **14** and **15**, as well as in other systems [50, 51, 52]. The poly(pyridyl)borates in **13–16** adopt *κ* ^2^ coordination mode as they leverage two pyridyl‐nitrogen donor sites to bind to rhodium. These structural outcome aligns with previous studies on [Me_2_B(Py)_2_]Rh(cod) as well as tris(pyrazolyl)borate ligand‐supported Rh‐(cod) complexes, where *κ* ^2^ bonding mode is commonly observed in the Rh(I), d^8^ metal center [53, 54]. Some Rh(I) complexes, however, can also exhibit alternative coordination patterns such as *κ* ^3^ binding [55], or a combination of geometries within the unit cell, depending on steric interaction between ligand substituents and the diene coligands [55, 56, 57]. Increasing steric hindrance generally favors the formation of square‐planar structures [58, 59].

In the tris(pyridyl)ligand supported complexes **13** and **16**, two pyridyl arms coordinate to the Rh(I) center, while the third arm rotates around the B─C bond and lies nearly parallel to the rhodium coordination plane, resulting in a “type A” binding mode (see Supporting Information, Figure S1) [57, 60]. The Rh•••N(3) distances of 3.84 and 3.81 Å, respectively, for **13** and **16**, are too long to be considered a significant bonding interaction (for comparison, the sum of Bondi's van der Waals radii of Rh and N is 3.15 Å) [61]. The *ipso*‐C to Rh separation in the hanging pyridyl groups of **13** and **16** measures 3.32 and 3.11 Å, respectively. The shorter distance in **16** is likely due to nbd being smaller and more compact than cod, with cone angles of 166° and 186°, respectively [62], which allows a closer approach of the unbound pyridyl ring to the metal center. In complex **14**, which has a B‐phenylated, *κ * [2]‐bound tris(pyridyl) ligand, the uncoordinated pyridyl ring orients away from the metal center, while the phenyl group is positioned above the N─Rh─N plane occupying the space left vacant by the uncoordinated arm. This structural motif is not unusual for B‐phenylated ligands, as seen in [PhB(6‐(CF_3_)Py)_3_]Tl [45] and [PhB(3‐(CF_3_)Pz)_3_]Ag(C_2_H_4_) [63]. In the rhodium complex **15,** one of the phenyl groups of the B‐phenylated bis(pyridyl)borate is positioned above the N─Rh─N plane, similar to what is observed in **14**.

Selected structural parameters of **13–16** are presented in Table 1, along with several related species. There are no structurally characterized tris(pyridyl)borate rhodium complexes available for comparison, while one bis(pyridyl)borate Rh(I)‐cod complex has been reported. Overall, the Rh─N bond lengths, Rh─C (olefinic) bond lengths, C═C distances within the coordinated cyclooctadiene, and N─Rh─N angles in complexes [MeB(6‐(CF_3_)Py)_3_]Rh(cod) (**13**), [PhB(6‐(CF_3_)Py)_3_]Rh(cod) (**14**), and [Ph_2_B(6‐(CF_3_)Py)_2_]Rh(cod) (**15**) are comparable and fall within the range reported for related *κ* ^2^ complexes listed in the Table 1. It is also useful to analyze the steric effects of supporting ligands in compounds **13–15**. A quantitative approach to for this involves buried volume (%*V*_Bur_) calculations and steric maps using the SambVca 2.1 web application [64], in which the %*V*_Bur_ corresponds to the fraction of the volume of a sphere centered on the metal occupied by the coordinated ligand of focus, while the topographic steric map provides a graphical representation of the steric profile of a ligand using color‐coded contour maps. Analysis of the topographic steric maps of fluorinated supporting ligands using this method and X‐ray data reveals percent buried volume of 53.5%, 52.9%, and 52.6%, respectively (see Figure S15), indicating quite similar steric protection at the rhodium centers by the supporting ligands in **13**–**15**. Bond distances associated with rhodium of **16**, which has a smaller diene nbd, show no significant differences from the corresponding separations of **13**. It is also possible to estimate the steric bulk of the cyclic dienes, nbd and cod, in [MeB(6‐(CF_3_)Py)_3_]Rh(cod) (**13**), [MeB(6‐(CF_3_)Py)_3_]Rh(nbd) (**16**) using %*V*_Bur_ from X‐ray crystallographic data [65]. As expected, the nbd is smaller than cod, with %*V*_Bur_ of 42.2% vs. 47.2%, respectively.

**TABLE 1:** Selected bond lengths (Å) and angle (°) for Rh(I)‐cod complexes supported by *κ* ^2^‐tris and ‐bis(pyridyl)borate, *κ* ^2^‐tris and ‐bis(pyrazolyl)borate ligands. Italicized numbers indicate the values for the second molecule in the asymmetric unit.

Compound	Rh–N (Å)	Rh–C (Å)	C=C (Å)	∠N–Rh–N (°)	References
[MeB(6‐(CF_3_)Py)_3_]Rh(cod) (**13**)	2.1147(11), 2.1616(11)	2.1266(13)‐2.1522(13)	1.394(2), 1.3999(18)	83.70(4)	This work
[PhB(6‐(CF_3_)Py)_3_]Rh(cod) (**14**)	2.116(5), 2.122(5); *2.115(5), 2.125(5)*	2.122(6)‐2.144(6); *2.124(6)‐2.144(6)*	1.388(8), 1.381(10); *1.390(8), 1.397(9)*	85.65(18); *84.98(18)*	This work
[HB(3,5‐(*^i^* Pr)_2_Pz)_3_]Rh(cod)	2.099(3), 2.133(3)	2.126(4)‐2.141(4)	1.367(6), 1.378(6)	82.50(1)	[56]
[HB(3‐Ph‐5‐Me)Pz)_3_]Rh(cod)	2.0950(15), 2.1003(14)	2.1344(19)‐2.1457(17)	1.387(3), 1.391(3)	82.60(6)	[60]
[HB(3‐PhPz)_3_]Rh(cod)	2.094(6), 2.094(6)	2.122(8)‐2.145(8)	1.357(12), 1.377(12)	84.56(24)	[57]
[Ph_2_B(6‐(CF_3_)Py)_2_]Rh(cod) (**15**)	2.1186(15), 2.1332 (15)	2.1315(19)‐2.1415(19)	1.390(3), 1.395(3)	85.66(6)	This work
[Me_2_B(Py)_2_]Rh(cod)	2.1075(19), 2.108(2)	2.120(2)‐2.144(4)	1.380(4), 1.399(4)	85.59(7)	[49]
[MeB(6‐(CF_3_)Py)_3_]Rh(nbd) (**16**)	2.1178(13), 2.1587(13)	2.1216(16)‐2.1365(16)	1.390(2), 1.394(2)	82.77(5)	This work
[HB(3,5‐(CF_3_)_2_Pz)_3_]Rh(nbd)	2.1248(11), 2.1523(10)	2.1145(13)‐2.1404(13)	1.3923(18), 1.3955(19)	84.98(4)	[34]
[HB(3‐PhPz)_3_]Rh(nbd)	2.089(3), 2.096(3)	2.080(4)‐ 2.129(4)	1.362(6), 1.362(7)	85.0(1)	[57]
[HB(3,5‐(*^i^* Pr)_2_Pz)_3_]Rh(nbd)	2.118(4), 2.133(4)	2.097(6)‐ 2.119(6)	1.368(10), 1.387(10)	85.02(14)	[56]

^1^H and ^13^C NMR spectra of complexes [MeB(6‐(CF_3_)Py)_3_]Rh(cod) (**13**) and [PhB(6‐(CF_3_)Py)_3_]Rh(cod) (**14**) at room temperature reveal two sets of resonances corresponding to inequivalent pyridyl rings in a 1:2 ratio. Most of the reported rhodium(I)‐diene complexes supported by pyrazolyl‐based ligands exhibit fluxional behavior on the NMR timescale at room temperature, which can be slowed down sufficiently at low temperatures to distinguish coordinated and uncoordinated pyrazolyl rings [49]. However, such fluxionality at room temperature is not universal, as some rhodium(I)‐cod complexes with tris(pyrazolyl)borate also show no exchange at room temperature [55, 66]. The exchange rate is highly influenced by steric congestion around the metal center. Given that pyridyl‐based ligands are more sterically demanding than their pyrazolyl counterparts [45], the observed inequivalence of the pyridyl rings in **13** and **14** is likely due to restricted ring exchange. In most Rh–cod complexes, the olefinic protons and carbons of the cod ligand appear equivalent, likely due to rapid fluxionality or the overall molecular symmetry [62, 67]. In complexes **13–15**, the cod double bonds are oriented perpendicular to an aromatic ring positioned above them (a pyridyl ring in **13** and phenyl rings in **14** and **15**). This arrangement introduces ring current effects that render the olefinic signal nonequivalent. For example, the cod olefinic protons in **13**, **14**, and **15** resonate at *δ* 3.87/2.98, 3.84/2.80, and 3.83/2.82 ppm, respectively, with the corresponding carbons appearing as doublets at *δ* 82.0/80.0, 82.8/80.5, and 82.9/80.1 ppm. Interestingly, the ^19^F spectra of **14** and **15** reveal delicate doublets, arising from through‐space coupling between rhodium and fluorines, with small coupling constants of about 1.2–1.3 Hz. Compound **16** was synthesized to investigate the effect of coligands (cod vs. nbd) on the fluxional behavior of complexes in solution and subsequently, to provide a direct steric comparison with a previously reported catalyst [HB(3,5‐(CF_3_)_2_Pz)_3_]Rh(nbd) [34]. Compound **16**, which has the smaller olefin, nbd, exhibits fluxional behavior in solution as evident from the ^1^H and ^13^C{^1^H} NMR spectra at room temperature, rendering the coordinated and uncoordinated pyridyl rings equivalent on the NMR time scale due to fast exchange. The broad olefinic proton signals from the nbd moiety further support this dynamic process (see Figure S11), as they are likely affected by the supporting ligand coordination modes. Upon cooling to −10°C, the exchange slows sufficiently to yield well‐resolved peaks; for example, the nbd olefinic protons appear at *δ* 3.87 and 3.08 ppm, and the coordinated and uncoordinated pyridyl rings become distinguishable.

**FIGURE 4:**
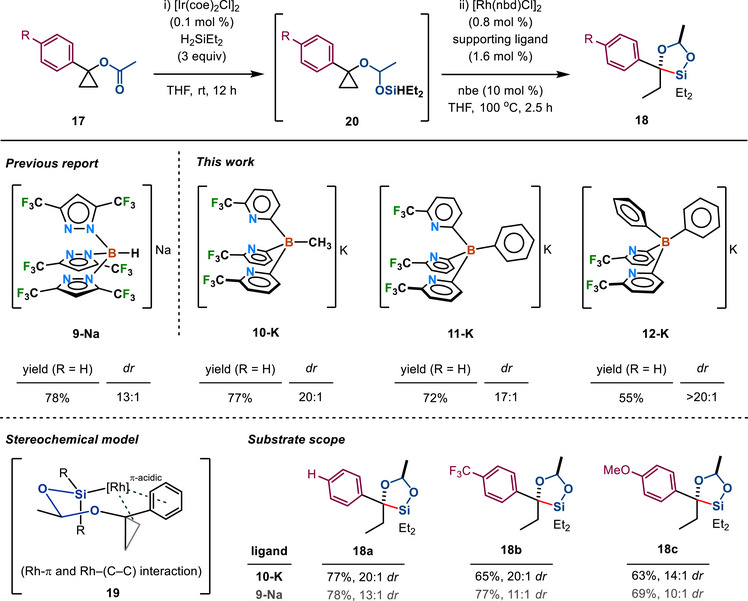
Rhodium‐catalyzed umpolung α‐selective C─C silylation of cyclopropyl acetates using acetal directing groups, and reaction yields by varying ligands.

The ability of fluorinated tris‐ and bis‐(pyridyl)borate ligands to serve as effective ligands for rhodium(I) prompted us to examine their properties in greater detail. The flexibility in supporting ligand coordination modes and possible variations in *η* ^4^‐*η* ^2^ hapticity offer interesting options for catalytic processes. Using the previously optimized conditions, we tested electronically and sterically different poly(pyridyl)borate ligands **10–12** for reactivity and selectivity in Rh‐catalyzed C─C silylation of three electronically varied cyclopropyl acetates (Figure 4). Encouragingly, the reaction of **17a** using ligand **10**, bearing an electron‐withdrawing 6‐CF_3_‐substituted pyridyl group, delivered the umpolung product, dioxasilolane **18a** in 77% yield, comparable to 78% yield obtained with [HB(3,5‐(CF_3_)_2_Pz)_3_]Rh(nbd) involving the highly fluorinated tris(pyrazolyl)borate ligand **9** [34], but with a notably improved diastereoselectivity of 20:1 (cf., 13:1 *dr* with **9**). Importantly, no β‐functionalized product (e.g., **4**) was observed [34]. Furthermore, electronically deactivated and activated cyclopropyl acetates **17b** and **17c** successfully underwent α‐selective C─C silylation with **10‐K**, affording **18b** and **18c** with significantly improved diastereoselectivities, albeit with slightly diminished yields. For **18b**, a 20:1 *dr* was achieved (cf., 11:1 *dr* using **9‐Na**), and for **18c**, a 14:1 *dr* was obtained (cf., 10:1 *dr* using **9‐Na**).

This enhanced diastereoselectivity is attributed to the steric augmentation at rhodium catalyst ligated with **10‐K**, which facilitates a more favorable chair‐like transition‐state geometry that enhances Rh–π (arene) and Rh–(prochiral C─C) interactions within the catalytically active intermediate **19**. Analysis of the topographic steric map and percent buried volumes (%*V*_Bur_) of supporting ligands using SambVca [64] for compound **16** and [HB(3,5‐(CF_3_)_3_Pz)_3_]Rh(nbd) clearly indicates a significantly greater steric encumbrance around the metal center in compound **16** (56.4% vs. 51.9%; Figure S16). Substituting the methyl group at boron with a phenyl substituent (i.e., the use of ligand **11**) resulted in a modest decrease in both yield (72%) and diastereoselectivity (17:1 *dr*). Lastly, **12**, featuring a less‐fluorinated bis(pyridyl)borate framework, led to a somewhat sluggish reaction, affording 55% yield but with uncompromised diastereoselectivity. This outcome is presumably due to substitution of a more electron‐withdrawing pyridyl ring with a moderately electron‐donating phenyl group (*σ*_p_ = 0.17 vs. −0.01, respectively), while maintaining a comparable steric environment at the rhodium site [68].

**FIGURE 5:**
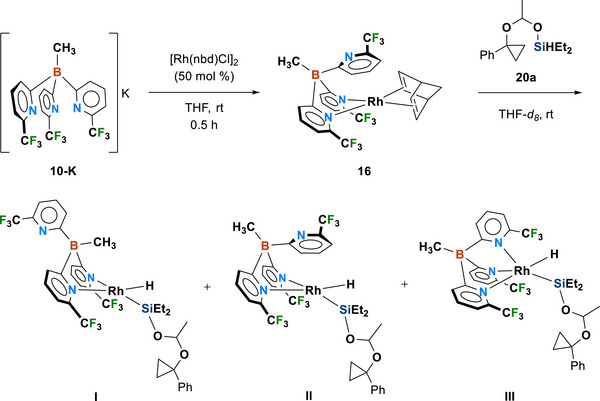
Likely Rh hydride species that could result in rhodium‐catalyzed C─C silylation directed by silyl acetals (see Figure S18 for ^1^H NMR data).

With the improved reactivity and stereoselectivity observed upon employing poly(pyridyl)borate ligands in Rh‐catalyzed C─C silylation directed by silyl acetals, we next sought to identify mechanistically relevant organometallic species using ^1^H NMR spectroscopy. At *t* = 1 min, ^1^H NMR spectrum revealed multiple Rh─H species, likely arising from oxidative addition of cyclopropyl hydrosilyl acetal (**20**, see Figure 5) to [MeB(6‐(CF_3_)Py)_3_]Rh(nbd) (**16**), at *δ* −13.35 (d, *J* = 21.3 Hz), −13.47 (d, *J* = 23.5 Hz), −13.50 (d, *J* = 14.7 Hz), −13.62 (s), and −13.74 (s) ppm (see Figure S18) [11, 12, 13, 69, 70]. The doublets suggest the presence of either two *κ* ^2^‐bonded tris(pyridyl)borate structures (**I** and **II**, with type B and A supporting ligand coordination modes illustrated in Figure S1) or one *κ* ^3^‐bonded tris(pyridyl)borate structure **III** (involving the type C supporting ligand coordination mode), which likely represent resting states of the hydrogenative C─C silyl insertion (Figure 5). Interestingly, three Rh─H doublets within a narrow ca. 1 ppm window indicate that the distinct coordinating modes in I, II, and III exert only subtle effects on the overall chemical environment of the rhodium species. Over the course of 100 min, the doublet at *δ* −13.35 ppm disappears rapidly, while the singlet at *δ* −13.62 ppm fades more gradually. The broad singlets are attributed to Rh─H species exhibiting fluxional behavior (i.e., rapid interconversion between *κ* ^2^ and *κ* ^3^) at room temperature, leading to the ^1^ *J*(Rh─H) coupling unresolved [71, 72]. The remaining doublets gradually coalesce into singlets over time. The singlets observed at *δ* = −13.62 and −13.74 ppm thus correspond to rapidly exchanging, short‐lived Rh─H species whose dynamic behavior causes line broadening and apparent singlet character.

## 3 Conclusions

Rhodium complexes supported by fluorinated poly(pyridyl)borates featuring trifluoromethyl substituents catalyzed redox‐neutral C─C silylation of cyclopropyl acetates effectively, affording the umpolung products, dioxasilolanes, in good yield with a notably improved diastereoselectivity. The weakly donating nature of the fluorinated ligands and the relatively high steric demand of the poly(pyridyl)borates contribute to the high yields and selectivity. We also isolated and thoroughly characterized four new and quite rare poly(pyridyl)borate ligand‐supported rhodium(I) complexes, as their 1,5‐cyclooctadiene and norbornadiene adducts. Crystal structures reveal that in [MeB(6‐(CF_3_)Py)_3_]Rh(cod), [MeB(6‐(CF_3_)Py)_3_]Rh(nbd), [PhB(6‐(CF_3_)Py)_3_]Rh(cod), and [Ph_2_B(6‐(CF_3_)Py)_2_]Rh(cod), both tris‐ and bis‐(pyridyl)borate ligands bind to rhodium centers in a *κ* ^2^‐fashion utilizing two pyridyl donor arms, while the cyclic dienes display *η* ^4^‐coordination. In Rh‐cod complexes, solution NMR data at room temperature are consistent with expectations based on solid‐state structures. The Rh‐nbd complex with the smaller dialkene shows fluxional behavior in solution at room temperature. The findings reported in this work highlight the potential of these poly(pyridyl)borate ligands in metal coordination chemistry and demonstrate their capacity to influence regio‐ and diastereoselectivity in metal‐mediated processes, opening new possibilities for the development of catalytic methods in organic synthesis.

## 4 Experimental Section

### 4.1 General Information

All preparations and manipulations were carried out under an atmosphere of purified nitrogen using standard Schlenk techniques or in an MBraun drybox equipped with a −25°C refrigerator. Dichloromethane and hexane were dried by passing HPLC grade solvent through a Solvent Purification System (SPS, innovative technologies inc.) and stored in Straus flasks. Glassware was oven dried overnight at 150°C. Solvents were purchased from commercial sources and purified before use. NMR spectra were recorded at 25°C on a JEOL Eclipse 400 spectrometer (^1^H, 400 MHz; ^13^C, 101 MHz, ^19^F, 376 MHz) or on a JEOL Eclipse 500 (^1^H, 500 MHz; ^13^C, 126 MHz; ^19^F, 471 MHz). All the spectral data were collected at room temperature (unless noted) and processed on MNova. ^1^H and ^13^C NMR spectra are referenced to the solvent peak (^1^H, CDCl_3_ *δ* 7.26; ^13^C, CDCl_3_ *δ* 77.16; ^1^H, CD_2_Cl_2_ *δ* 5.32; CD_2_Cl_2_ *δ* 54.00). ^19^F NMR values were referenced to external CFCl_3_. ^1^H, ^13^C, and ^19^F NMR chemical shifts are reported in ppm and coupling constants (*J*) are reported in Hertz (Hz). Abbreviations used for signal assignments: Py = pyridyl, s = singlet, d = doublet, dd = doublet of doublet, t = triplet, q = quartet, m = multiplet, br = broad peak, brs = broad singlet, nbd = norbornadiene, cod = cyclooctadiene, nbdCH* = olefinic protons in nbd, nbd*C*H* = olefinic carbons in nbd. Elemental analyses were performed using a Perkin–Elmer Model 2400 CHN analyzer. High‐resolution (HR) mass spectra were recorded at Shimadzu Center Laboratory for Biological Mass Spectrometry at UTA. NMR solvents were purchased from Cambridge Isotopes Laboratories and used as received. [MeB(6‐(CF_3_)Py)_3_]K [73], [PhB(6‐(CF_3_)Py)_3_]K [45], [Ph_2_B(6‐(CF_3_)Py)_2_]K [47], [Rh(nbd)Cl]_2_ [74], [Rh(cod)Cl]_2_ [75], and 1‐phenyl cyclopropyl acetate [34] were synthesized as previously reported. All other reactants and reagents were purchased from commercial sources.

### 4.2 [MeB(6‐(CF_3_)Py)_3_]Rh(cod) **(13)**

[MeB(6‐(CF_3_)Py)_3_]K (100 mg, 0.199 mmol, 1 equiv.) and [Rh(cod)Cl]_2_ (49 mg, 0.099 mmol, 0.5 equiv.) were placed in a Schlenk flask and dissolved in anhydrous dichloromethane (10 mL). The resulting orange solution was allowed to stir for 3 h at room temperature and filtered through a bed of Celite. The solvent was removed under reduced pressure to obtain the product as an orange solid. X‐ray quality crystals of [MeB(6‐(CF_3_)Py)_3_]Rh(cod) were grown from dichloromethane/hexane at −20°C. Yield: 72% mg (82 mg, 0.143 mmol). ^1^H NMR (500 MHz, CDCl_3_): *δ* (ppm) 8.27 (d, *J* = 6.9 Hz, 2H, Py), 7.74 (t, *J* = 7.4 Hz, 2H, Py), 7.58‐7.55 (m, 2H, Py), 7.45 (d, *J* = 7.4 Hz, 2H, Py), 7.35 (d, *J* = 6.9 Hz, 1H, Py), 3.87 (bs, 2H, codC*H*), 2.98 (bs, 2H, codC*H*), 2.43 (bs, 2H, codC*H*_2_), 1.55 (bs, 2H, codC*H*_2_), 0.96 (m, 2H, codC*H*_2_), 0.63 (m, 2H, codC*H*_2_), 0.41 (s, 3H, B‐C*H*_3_). ^13^C{^1^H} NMR (126 MHz, CDCl_3_): *δ* (ppm) 187.7 (q, ^1^ *J*_B‐C_ = 56.6 Hz, B‐Py), 147.6 (q, ^2^ *J*_F‐C_ = 22.4 Hz, *C*‐CF_3_), 134.5 (Py), 134.4 (Py), 134.2 (Py), 128.8 (Py), 122.7 (q, ^1^ *J*_F‐C_ = 275.3 Hz, *C*F_3_), 122.1 (q, ^1^ *J*_F‐C_ = 277.7 Hz, *C*F_3_), 120.8 (Py), 114.7 (Py), 82.0 (d, ^1^ *J*_Rh‐C_ = 13.2 Hz, cod*C*H), 80.0 (d, ^1^ *J*_Rh‐C_ = 13.2 Hz, cod*C*H), 29.9 (s, cod*C*H_2_), 28.5 (s, cod*C*H_2_), 19.7 (q, ^1^ *J*_B‐C_ = 37.2 Hz, B‐*C*H_3_). ^19^F NMR (297 MHz, CDCl_3_): *δ* (ppm) −60.65 (s, 6F), −67.91 (s, 3F). Anal. Calc. C_27_H_24_BF_9_N_3_Rh: C, 47.18; H, 3.29; N, 6.33%. Found: C, 46.98; H, 3.45; N, 6.07%.

### 4.3 [PhB(6‐(CF_3_)Py)_3_]Rh(cod) (**14)**

[PhB(6‐(CF_3_)Py)_3_]K (120 mg, 0.212 mmol, 1 equiv.) and [Rh(cod)Cl]_2_ (52 mg, 0.106 mmol, 0.5 equiv.) were placed in a Schlenk flask and dissolved in anhydrous dichloromethane (10 mL). The resulting orange solution was allowed to stir for 3 h at room temperature and filtered through a bed of Celite. The solvent was removed under reduced pressure to obtain the product as an orange solid. X‐ray quality crystals of [PhB(6‐(CF_3_)Py)_3_]Rh(cod) were grown from dichloromethane/hexane at −20°C. Yield: 61% mg (82 mg, 0.129 mmol). ^1^H NMR (500 MHz, CD_2_Cl_2_): *δ* (ppm) 7.84 (d, *J* = 7.7 Hz, 1H, Py), 7.78 (t, *J* = 7.7 Hz, 1H, Py), 7.56 – 7.53 (m, 5H, Py), 7.46 (dd, *J* = 6.3, 2.6 Hz, 2H, Py), 6.99 (m, 3H, Ph), 6.32 (s, 2H, Ph), 3.84 (m, 2H, codC*H*), 2.80 (m, 2H, codC*H*), 2.46 – 2.43 (m, 2H, codC*H*_2_), 1.60 – 1.59 (m, 2H, codC*H*_2_), 1.02 (m, 2H, codC*H*_2_), 0.69 – 0.66 (m, 2H, codC*H*_2_). ^13^C{^1^H} NMR (126 MHz, CD_2_Cl_2_): *δ* (ppm) 188.0 (q, ^1^ *J*_B‐C_ = 56.8 Hz, B‐Py), 162.3 (q, ^1^ *J*_B‐C_ = 49.0 Hz, B‐Ph), 148.0 (q, ^2^ *J*_F‐C_ = 34.9 Hz, *C*‐CF_3_), 147.4 (q, ^2^ *J*_F‐C_ = 32.6 Hz, *C*‐CF_3_), 138.1 (s, Ph), 135.3 (s, Py), 134.0 (s, Py), 131.5 (s, Py), 128.7 (s, Py), 127.5 (s, Ph), 125.0 (s, Ph), 123.2 (q, ^1^ *J*_F‐C_ = 274.6 Hz, *C*F_3_), 122.6 (q, ^1^ *J*_F‐C_ = 272.4 Hz, *C*F_3_), 121.3 (s, Py), 116.1 (s, Py), 82.8 (d, ^1^ *J*_Rh‐C_ = 12.8 Hz, cod*C*H), 80.5 (d, ^1^ *J*_Rh‐C_ = 13.2 Hz, cod*C*H), 30.3 (s, cod*C*H_2_), 29.1 (s, cod*C*H_2_). ^19^F NMR (471 MHz, CD_2_Cl_2_): *δ* (ppm) –61.11 (d, *J*_Rh‐F_ = 1.2 Hz, 6F), −68.21 (s, 3F). HR‐MS [ESI, positive ion mode ESI‐TOF]: *m*/*z* for C_32_H_26_BF_9_N_3_Rh [M + H]^+^ calcd 738.1209, found 738.1230.

### 4.4 [Ph_2_B(6‐(CF_3_)Py)_2_]Rh(cod) (**15**)

[Ph_2_B(6‐(CF_3_)Py)_2_]K (100 mg, 0.201 mmol, 1 equiv.) and [Rh(cod)Cl]_2_ (50 mg, 0.100 mmol, 0.5 equiv.) were placed in a Schlenk flask and dissolved in anhydrous dichloromethane (10 mL). The resulting orange solution was allowed to stir for 3 h at room temperature and filtered through a bed of Celite. The solvent was removed under reduced pressure to obtain the product as an orange solid. X‐ray quality crystals of [Ph_2_B(6‐(CF_3_)Py)_2_]Rh(cod) were grown from dichloromethane/hexane at −20°C. Yield: 73% mg (98 mg, 0.147 mmol). ^1^H NMR (400 MHz, CD_2_Cl_2_): *δ* (ppm) 7.56‐7.50 (m, 4H, {Ph‐2H; Py‐2H}), 7.44 (dd, *J* = 7.6, 1.5 Hz, 2H, Py), 7.38 – 7.33 (m, 4H, {Ph‐2H; Py‐2H}), 7.24 (t, *J* = 7.3 Hz, 1H, Ph), 6.97 (m, 3H, Ph), 6.45 (brs, 2H, Ph), 3.83 (m, 2H, codC*H*), 2.82 (m, 2H, codC*H*), 2.45 – 2.41 (m, 2H, codC*H*_2_), 1.60 – 1.58 (m, 2H, codC*H*_2_), 0.94 – 0.92 (m, 2H, codC*H*_2_), 0.84 – 0.81 (m, 2H, codC*H*_2_). ^13^C{^1^H} NMR (100 MHz, CD_2_Cl_2_): *δ* (ppm) 189.5 (q, ^1^ *J*_B‐C_ = 57.1 Hz, B‐Py), 155.2 (q, ^2^ *J*_F‐C_ = 61.8 Hz, B‐Ph), 147.7 (br, *C*‐CF_3_), 137.4 (s, Ph), 135.9 (s, Ph), 134.7 (s, Py), 134.0 (s, Ph), 128.3 (s, Py), 127.2 (s, Ph), 124.8 (s, Ph), 124.7 (s, Ph), 122.7 (q, ^1^ *J*_F‐C_ = 275.2 Hz), 121.2 (s, Py), 82.9 (d, ^1^ *J*_Rh‐C_ = 12.8 Hz), 80.1 (d, ^1^ *J*_Rh‐C_ = 13.1 Hz), 30.3 (s, cod*C*H_2_), 29.1 (s, cod*C*H_2_). ^19^F NMR (376 MHz, CDCl_3_): *δ* (ppm) −61.21 (d, *J*_Rh‐F_ = 1.3 Hz). HR‐MS [ESI, positive ion mode ESI‐TOF]: *m*/*z* for C_32_H_29_BF_6_N_2_Rh [M + H]^+^ calcd 669.1383, found 669.1428.

### 4.5 [MeB(6‐(CF_3_)Py)_3_]Rh(nbd) (**16**)

[MeB(6‐(CF_3_)Py)_3_]K (150 mg, 0.298 mmol, 1 equiv.) and [Rh(nbd)Cl]_2_ (68.4 mg, 0.148 mmol, 0.5 equiv.) were placed in a Schlenk flask and dissolved in anhydrous dichloromethane (10 mL). The resulting orange solution was allowed to stir for 3 h at room temperature and filtered through a bed of Celite. The solvent was removed under reduced pressure to obtain the product as an orange solid. X‐ray quality crystals of [MeB(6‐(CF_3_)Py)_3_]Rh(nbd) were grown from dichloromethane/hexane at −20°C. Yield: 48% (94 mg, 0.143 mmol). ^1^H NMR (500 MHz, CDCl_3_, −10°C): *δ* (ppm) 8.16 (d, *J* = 6.5 Hz, 2H, Py), 7.67‐7.62 (m, 3H, Py), 7.41‐7.40 (m, 3H, Py), 7.31 (br, 1H, Py), 3.87 (br, 2H, nbdCH*), 3.64 (br, 1H, nbdC*H*), 3.08 (br, 2H, nbdCH*), 1.95 (br, 1H, nbdC*H*), 0.79 (s, 2H, nbdC*H*_2_), 0.53 (s, 3H, B‐C*H*_3_). ^13^C{^1^H} NMR (126 MHz, CDCl_3_, −10°C): *δ* (ppm) 186.8 (q, ^1^ *J*_C‐B_ = 53.6 Hz, Py), 147.1 (q, ^2^ *J*_C‐F_ = 31.4 Hz, *C*‐CF_3_), 135.0 (Py), 133.8 (s, Py), 133.3 (s, Py), 130.2 (s, Py), 121.8 (q, ^1^ *J*_F‐C_ = 273.1 Hz, *C*F_3_), 119.7 (s, Py), 114.8 (s, Py), 61.6 (d, ^2^ *J*_Rh‐C_ = 5.6 Hz, nbd*C*H), 59.4 (d, ^1^ *J*_Rh‐C_ = 10.2 Hz, nbd*C*H*), 54.0 (d, ^1^ *J*_Rh‐C_ = 11.3 Hz, nbd*C*H*), 49.3 (s, nbd*C*H_2_), 17.5 (q, ^1^ *J*_B‐C_ = 37.8 Hz, B‐*C*H_3_). ^19^F NMR (273 MHz, CDCl_3_, −10°C): *δ* (ppm) −62.87 (s, 6F), −68.13 (s, 3F). Anal. Calc. C_26_H_20_BF_9_N_3_Rh: C, 47.38; H, 3.06; N, 6.37%. Found: C, 46.98; H, 2.81; N, 6.29%.

### 4.6 C─C Silylation of Cyclopropyl Acetate

All reactions on the 0.2 mmol scale were performed following a previously reported procedure [34]. To a flame‐dried vial containing a mixture of [Ir(coe)_2_Cl]_2_ (0.1 mol %) and 1‐phenyl cyclopropyl acetate **17** (0.2 mmol, 1 equiv.) in THF (0.6 mL) at room temperature, diethylsilane (3 equiv.) was added in one portion. The vial was closed using a screw cap equipped with a Teflon liner. The reaction mixture was stirred for 12 h at room temperature. The volatiles were removed *in vacuo* to afford the silyl acetal **20**, which was directly used for a subsequent reaction without further purification.

To the vial containing the crude silyl acetals **20** (0.2 mmol), [Rh(nbd)Cl]_2_ (0.8 mol %), potassium salt of the ligand (1.6 mol %), and norbornene (10 mol %), THF (0.6 mL) were added. The vial was closed using a screw cap equipped with a Teflon liner and then heated at 100°C for 2.5 h. Reaction yields were determined by ^1^H NMR using mesitylene as an internal standard, and the *dr* was determined by GC‐MS spectrometry and ^1^H NMR spectroscopy [34].

### 4.7 Study of Reaction Intermediates

To a flame‐dried vial containing [Rh(nbd)Cl]_2_ (10 mol%) and [MeB(6‐(CF_3_)Py)_3_]K (**10‐K**, 20 mol %)_,_ THF (0.5 mL) was added. The vial was capped with a Teflon‐lined screw cap, and the resulting solution was stirred at room temperature for 30 min. The volatile was evaporated by placing the reaction mixture directly under vacuum for 0.5 h. The catalyst **16** was then sequentially treated with silyl acetal **20a** (59 mg, 0.2 mmol), norbornene (38 mg, 0.4 mmol, 2 equiv), and THF‐*d*_8_ (0.50 mL). This solution was transferred to a J‐Young NMR tube, and the reaction was monitored by ^1^H NMR spectroscopy.

### 4.8 X‐Ray Structure Determinations

A suitable crystal covered with a layer of hydrocarbon/Paratone‐N oil was selected and mounted on a Cryo‐loop, and immediately placed in the low temperature nitrogen stream. The X‐ray intensity data were measured at 100(2) K (unless otherwise noted) on a Bruker Smart ApexII or Bruker D8 Quest equipped with a PHOTON II 7 CPAD detector and an Oxford Cryosystems 700 series cooler, a graphite monochromator, and a Mo Kα fine‐focus sealed tube (λ = 0.71073 Å). Intensity data were processed, and appropriate absorption corrections were applied using the Bruker Apex program suite. Initial atomic positions were located by SHELXT [76], and the structures of the compounds were refined by the least‐squares method using SHELXL [77] within Olex2 GUI [78]. Compounds [MeB(6‐(CF_3_)Py)_3_]Rh(cod) (**13**), [MeB(6‐(CF_3_)Py)_3_]Rh(nbd) (**16**), and [Ph_2_B(6‐(CF_3_)Py)_2_]Rh(cod) (**15**) show no crystallographic issues. Crystals of [PhB(6‐(CF_3_)Py)_3_]Rh(cod) (**14**) show nonmerohedral twinning. It crystallizes in triclinic P‐1 space group. The Cell_Now within Bruker Apex6 was used to de‐twin the data into two components. Structure was solved and refined first using detwinned HKLF 4 data, and then final refinements were done using reflections from both domains (HKLF5 file) using Bruker Apex [79]. There are two molecules of **14** in the asymmetric unit. All the non‐hydrogen atoms in **13–16** were refined anisotropically. Hydrogen atoms of the olefinic carbons of the norbornadiene moiety of [MeB(6‐(CF_3_)Py)_3_]Rh(nbd) (**16**) were located on difference maps, included and refined freely. Remaining hydrogen atoms and those of **13–15** were included at calculated positions and refined using appropriate riding models. X‐ray structural figures were generated using Olex2.

## Conflicts of Interest

The authors declare no conflicts of interest.

## Supporting information



Additional experimental details, NMR spectra and data, analysis of topographic steric map, and references. CCDC Deposition Number(s) 2494597 (for **16**), 2494598 (for **13**), 2494599 (for **14**), 2494600 (for **15**) contain the supplementary crystallographic data for this paper. These data are provided free of charge by the joint Cambridge Crystallographic Data Centre and Fachinformationszentrum Karlsruhe http://www.ccdc.cam.ac.uk/structures">Access Structures service.

**Supporting File 1**: chem70722‐sup‐0002‐SuppMat.pdf.

**Supporting File 2**: chem70722‐sup‐0003‐SuppMat.cif.

## Data Availability

The data that support the findings of this study are available in the supplementary material of this article.;
