# Supplementary material for: Regio‐ and Diastereoselective C–C Silylation of Cyclopropyl Acetates Harnessing Fluorinated Poly(pyridyl)Borate Rhodium Catalysts
Source: Chemistry. 2026 Jan 24;32(14):e03217. doi: 10.1002/chem.202503217 (PMC13088001; doi:10.1002/chem.202503217)
Supplement: Supplementary file 1 — Additional experimental details, NMR spectra and data, analysis of topographic steric map, and references. CCDC Deposition Number(s) 2494597 (for 16), 2494598 (for 13), 2494599 (for 14), 2494600 (for 15) contain the supplementary crystallographic data for this paper. These data are provided free of charge by the joint Cambridge Crystallographic Data Centre and Fachinformationszentrum Karlsruhe http://www.ccdc.cam.ac.uk/structures">Access Structures service. [file CHEM-32-e03217-s003.pdf]

Supporting Information

Regio- and diastereoselective C–C silylation of cyclopropyl acetates harnessing fluorinated poly(pyridyl)borate rhodium catalysts

Vo Quang Huy Phan,† Suman Das Adhikary,† Junha Jeon,* H. V. Rasika Dias*

The Department of Chemistry and Biochemistry, The University of Texas at Arlington, Arlington, Texas 76019, USA

E-mail: junha.jeon@uta.edu, dias@uta.edu

†equal contributions

Table of contents:

Possible coordination mode in tris(pyridyl)borate rhodium(I) complexes 2

NMR spectroscopic data 2

Topographic steric maps 15

X-ray Data Collection and Structure Determinations 17

Catalysis 21

References 24

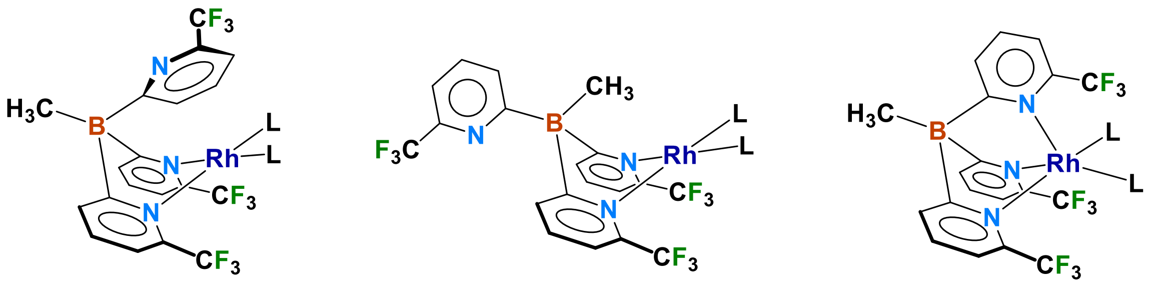

Type A | Type B | Type C

Figure S1. Three possible coordination modes in tris(pyridyl)borate rhodium(I) complexes, LL= 1,5-cyclooctadiene (cod)

NMR Spectroscopy

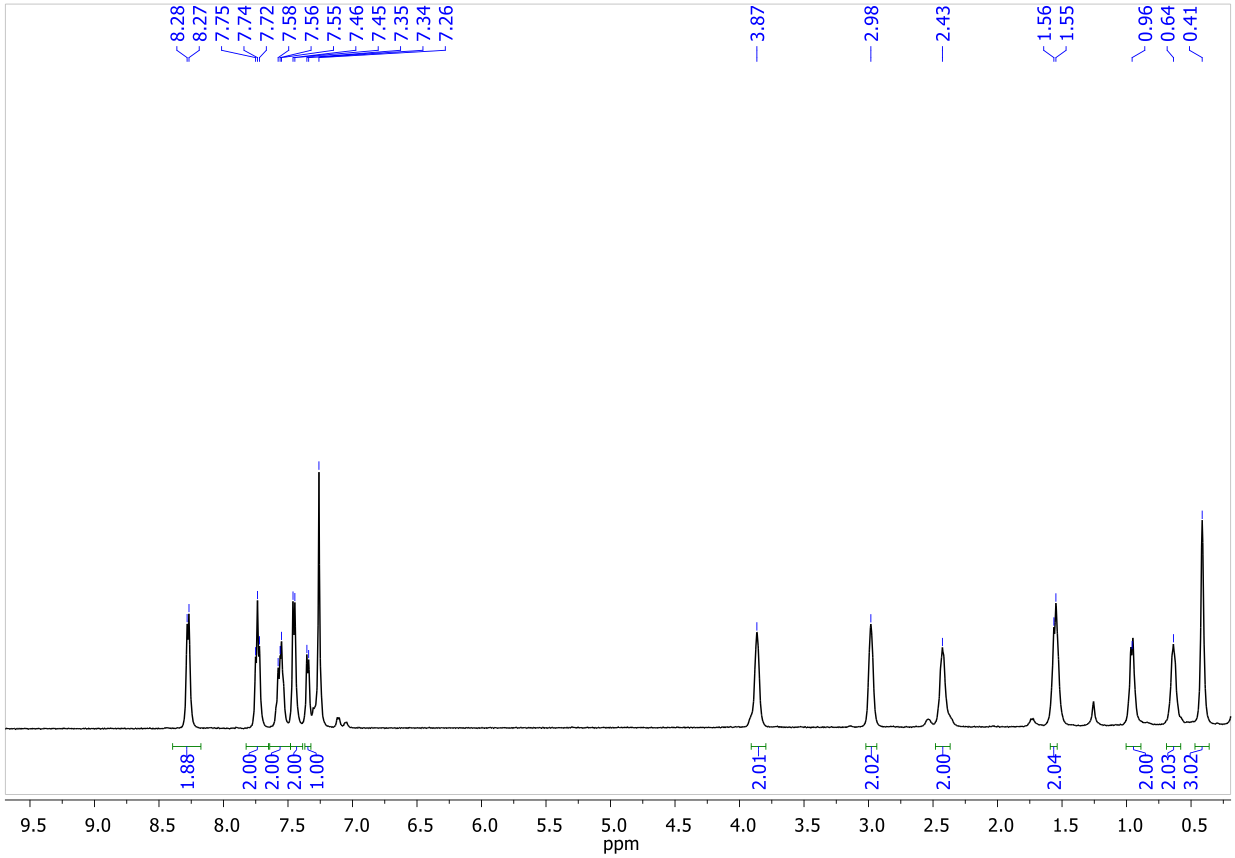

Figure S2. ^{1}H NMR of [MeB(6-(CF_3)Py)$_3$]Rh(cod) (**13**) in $CDCl_3$

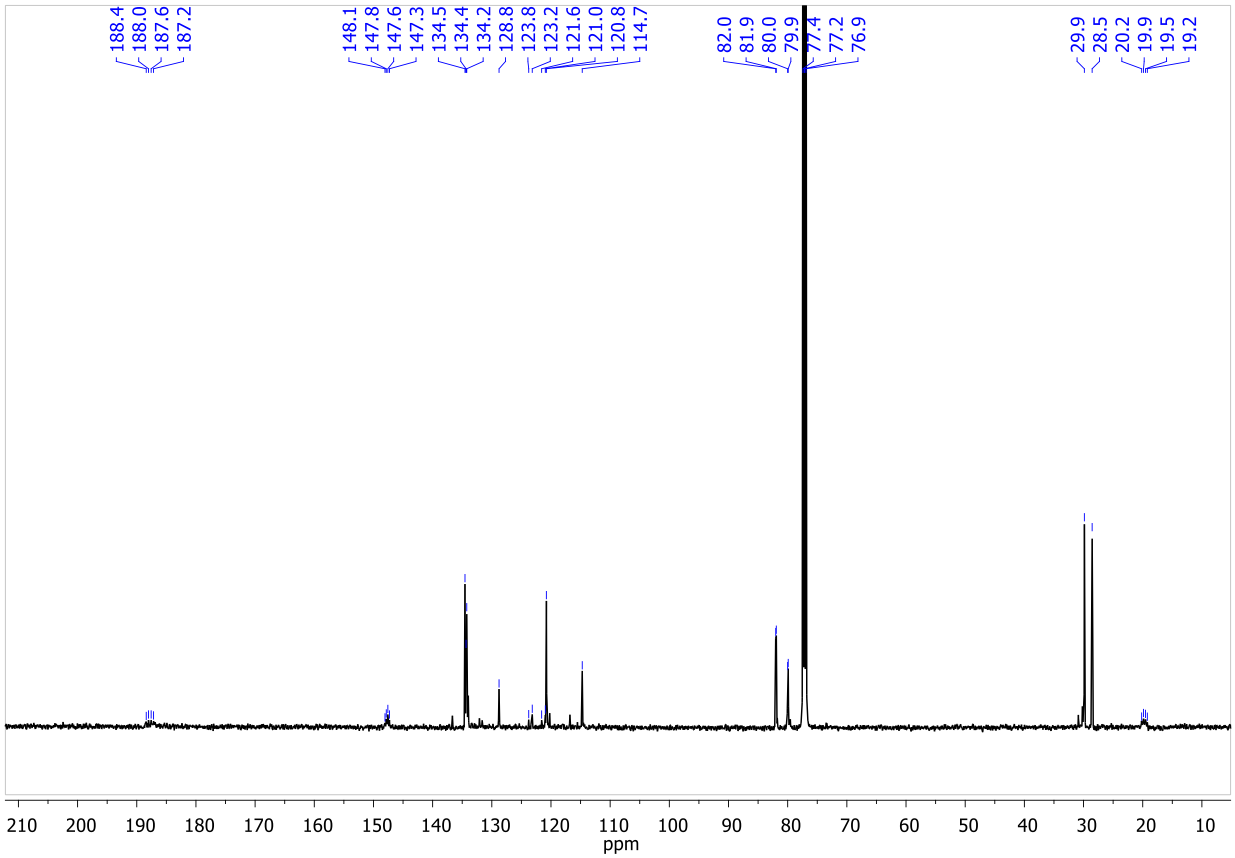

Figure S3. $^{13}C\{^1H\}$ NMR of [MeB(6-(CF_3)Py)$_3$]Rh(cod) (**13**) in $CDCl_3$

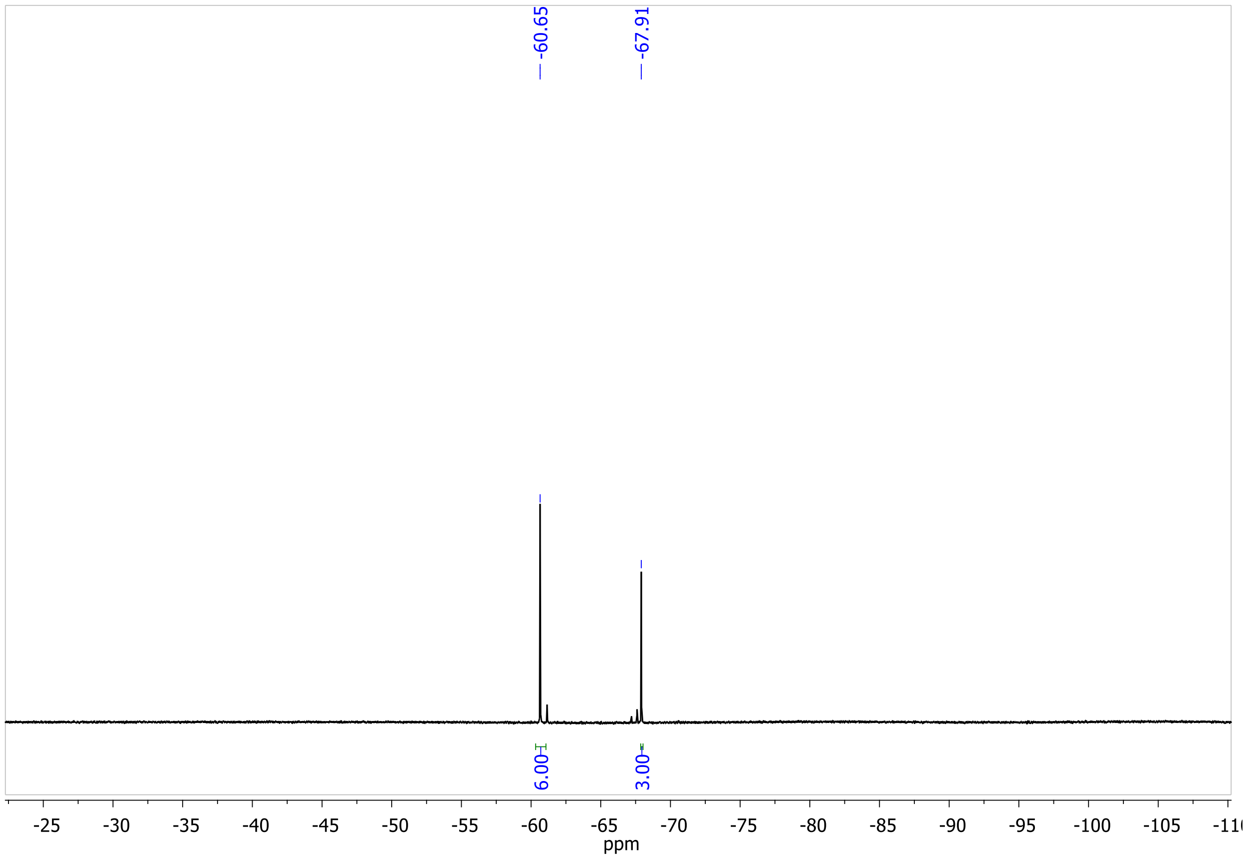

Figure S4. ^{19}F NMR of [MeB(6-(CF_3)Py)$_3$]Rh(cod) (**13**) in $CDCl_3$

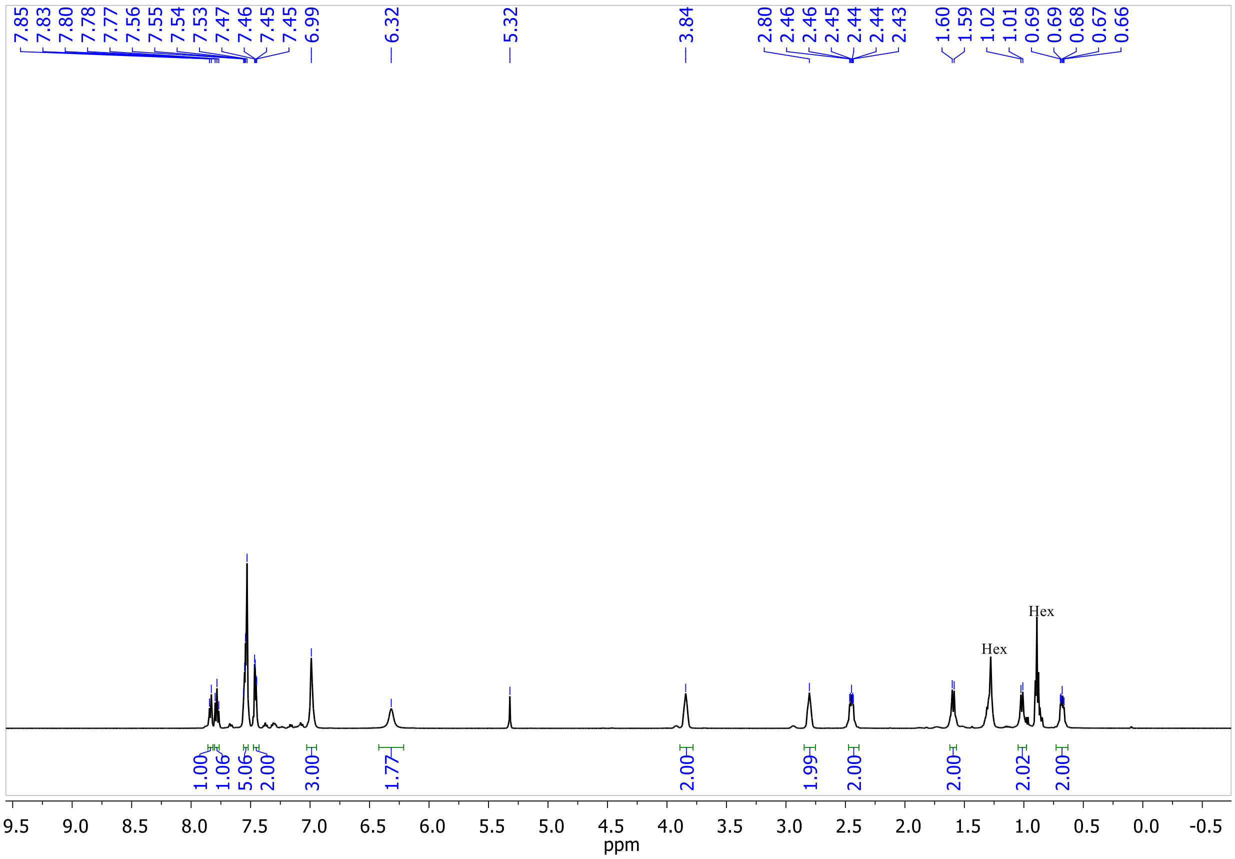

Figure S5. ^{1}H NMR of [PhB(6-(CF_3)Py)$_3$]Rh(cod) (**14**) in CD_2Cl_2

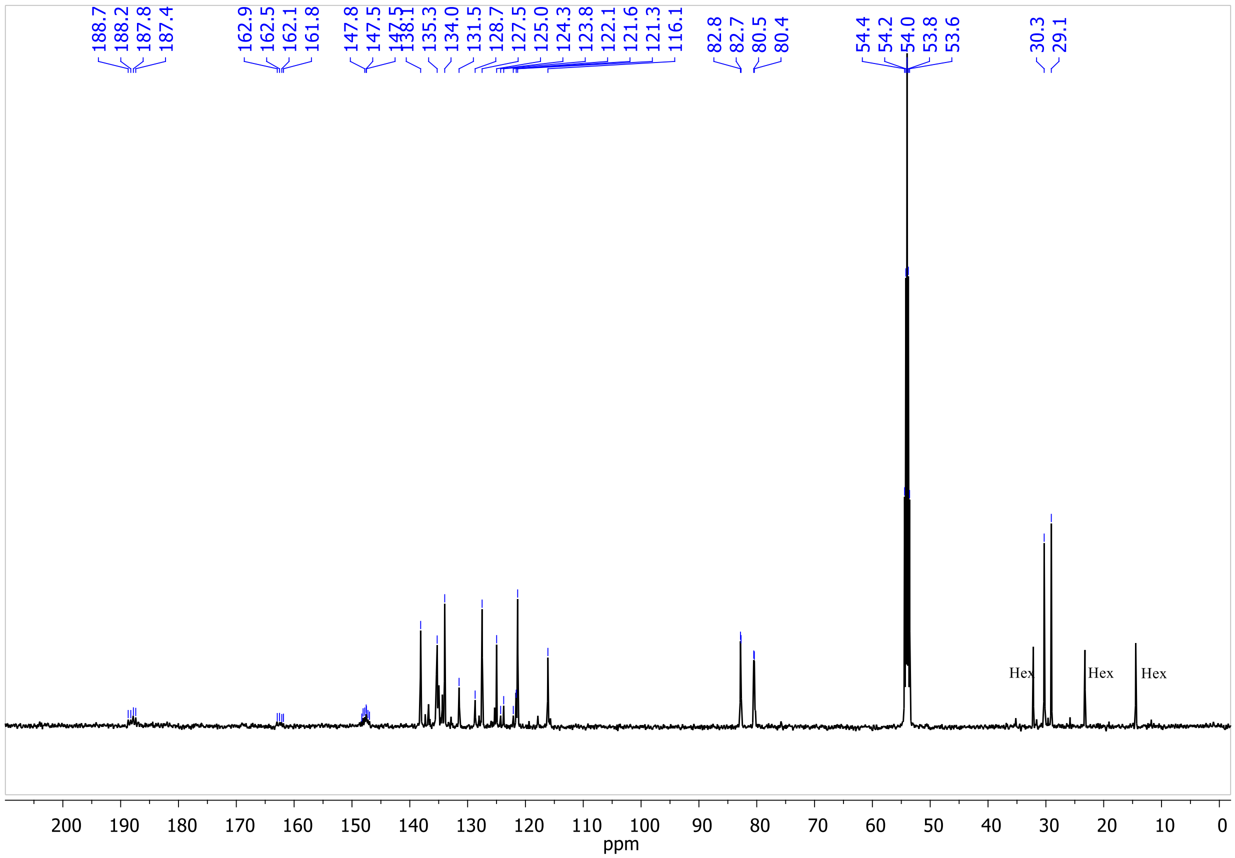

Figure S6. $^{13}C\{^1H\}$ NMR of $[PhB(6\text{-}(CF_3)Py)_3]Rh(cod)$ (**14**) in CD_2Cl_2

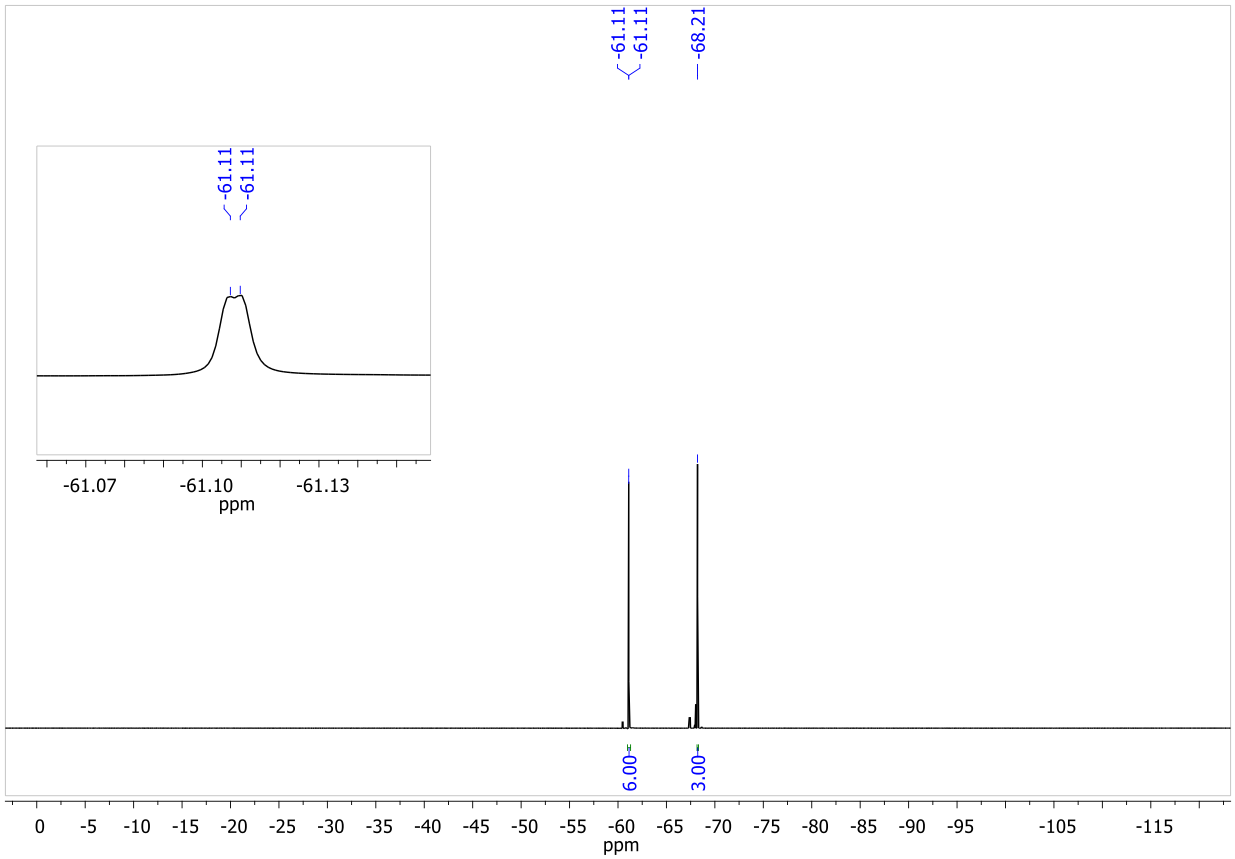

Figure S7. ^{19}F NMR of $[PhB(6\text{-}(CF_3)Py)_3]Rh(cod)$ (**14**) in CD_2Cl_2

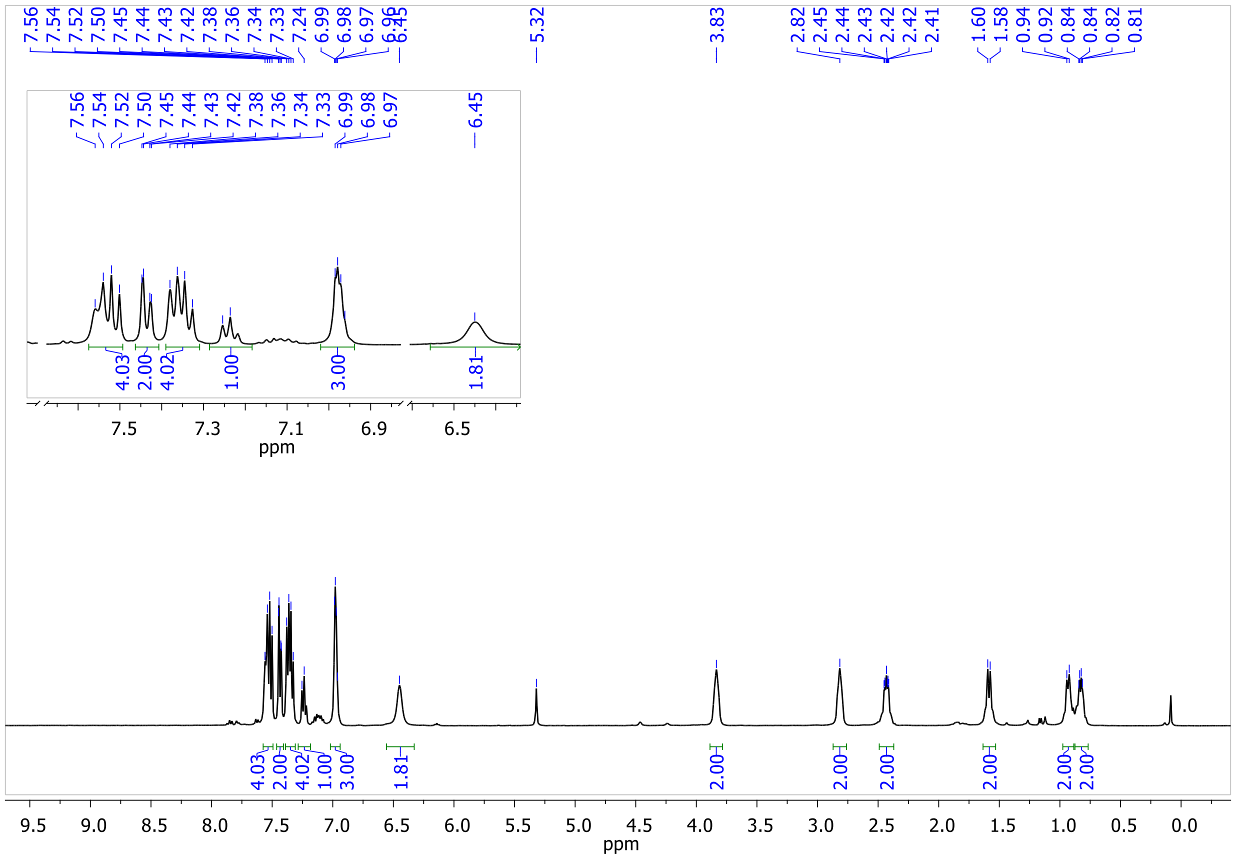

Figure S8. ^{1}H NMR of $[Ph_2B(6\text{-}(CF_3)Py)_2]Rh(cod)$ (**15**) in CD_2Cl_2

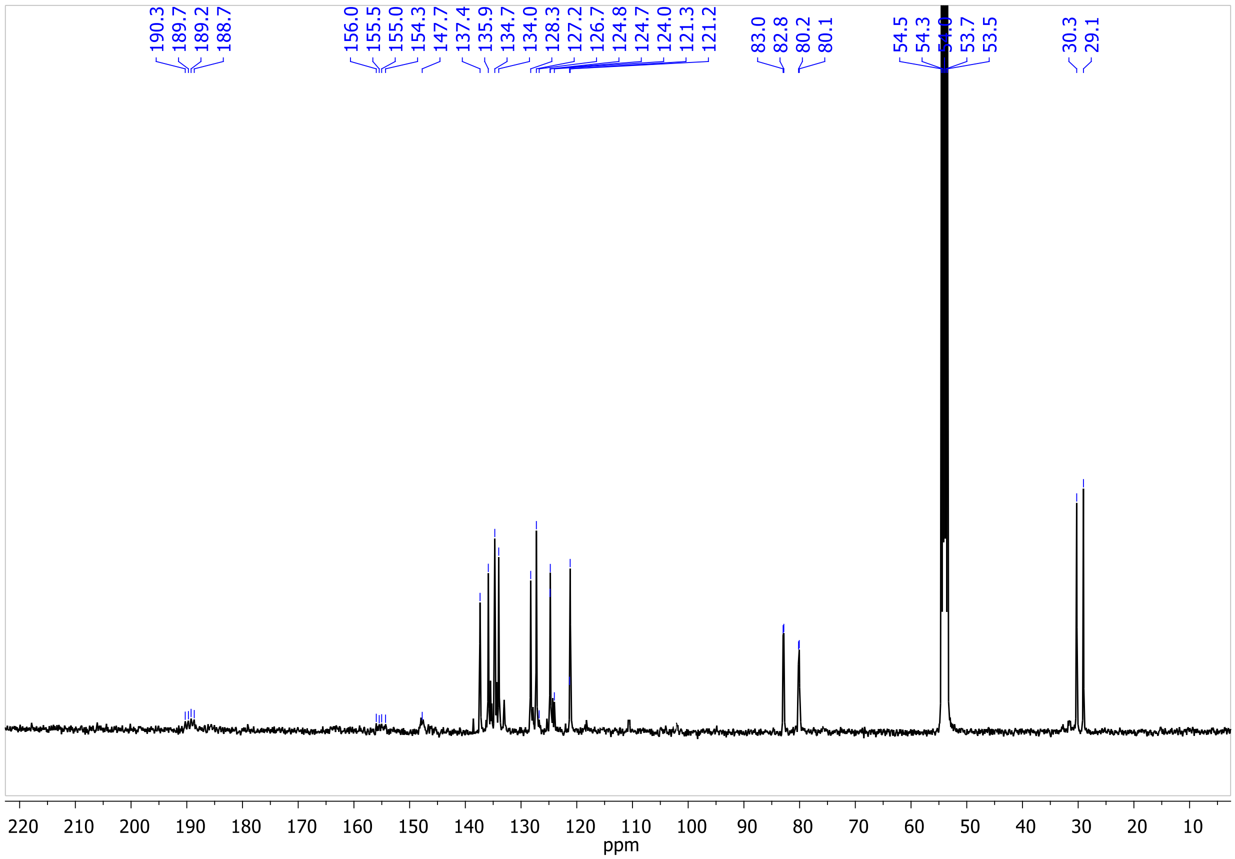

Figure S9. $^{13}C\{^{1}H\}$ NMR of $[Ph_2B(6\text{-}(CF_3)Py)_2]Rh(cod)$ (**15**) in CD_2Cl_2

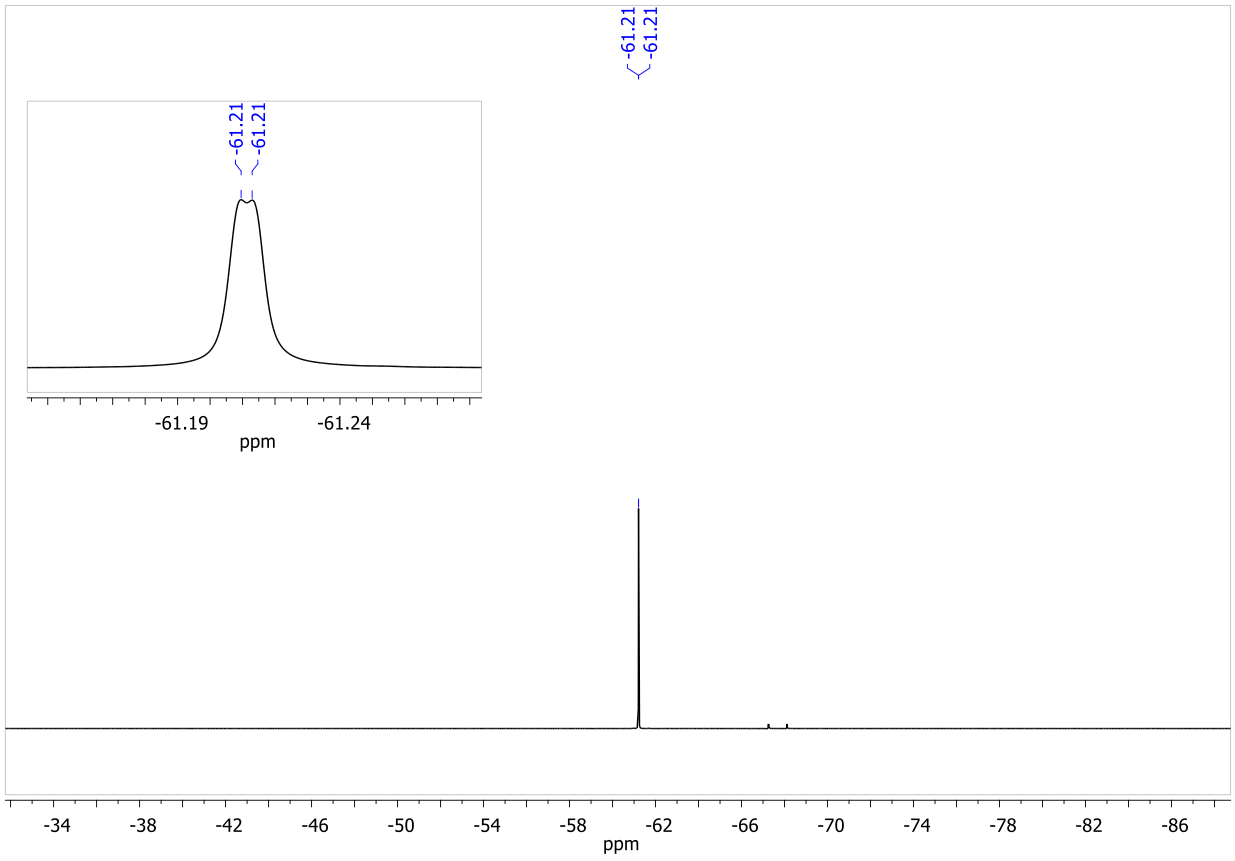

Figure S10. ^{19}F NMR of $[Ph_2B(6\text{-}(CF_3)Py)_2]Rh(cod)$ (**15**) in CD_2Cl_2

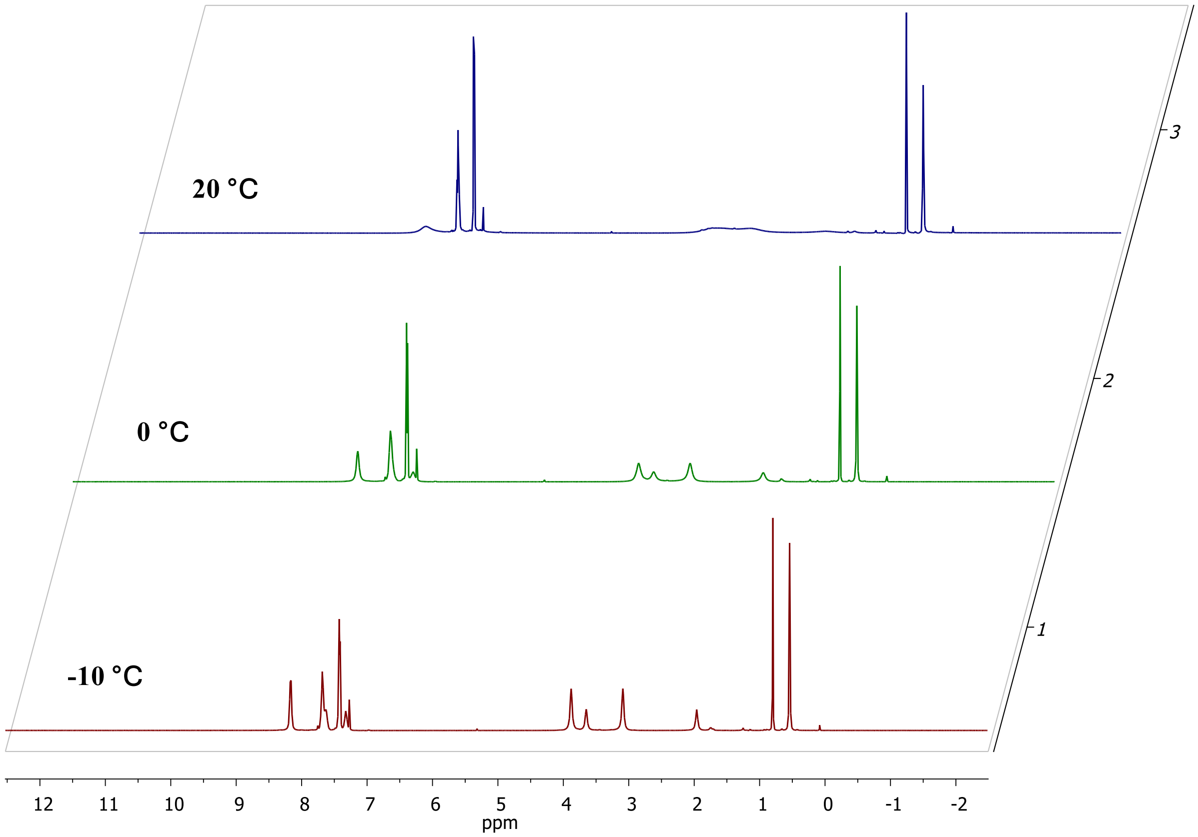

Figure S11. Variable temperature ^{1}H NMR spectra of [MeB(6-(CF_3)Py)$_3$]Rh(nbd) (**16**) in $CDCl_3$ from 20 °C to -10 °C

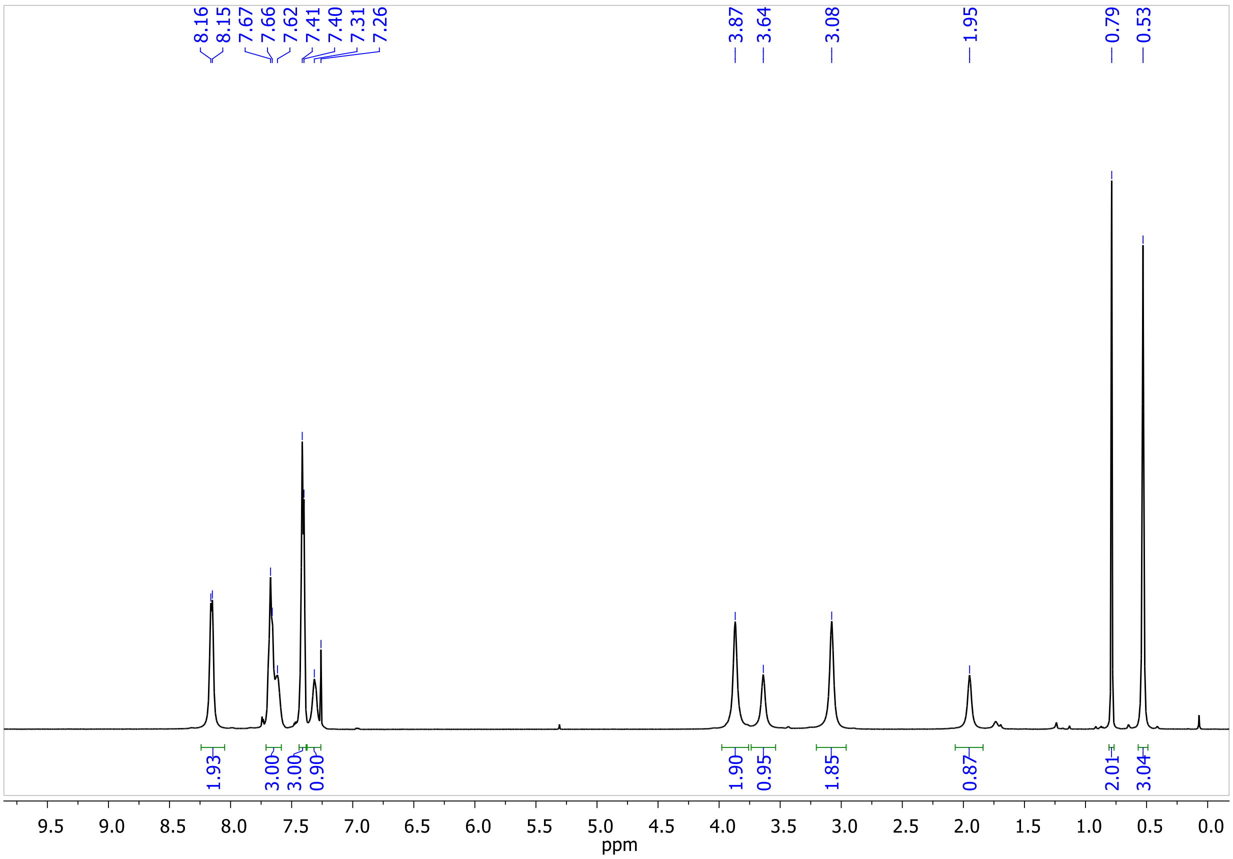

Figure S12. ^{1}H NMR of [MeB(6-(CF_3)Py)$_3$]Rh(nbd) (**16**) in $CDCl_3$ at -10 °C

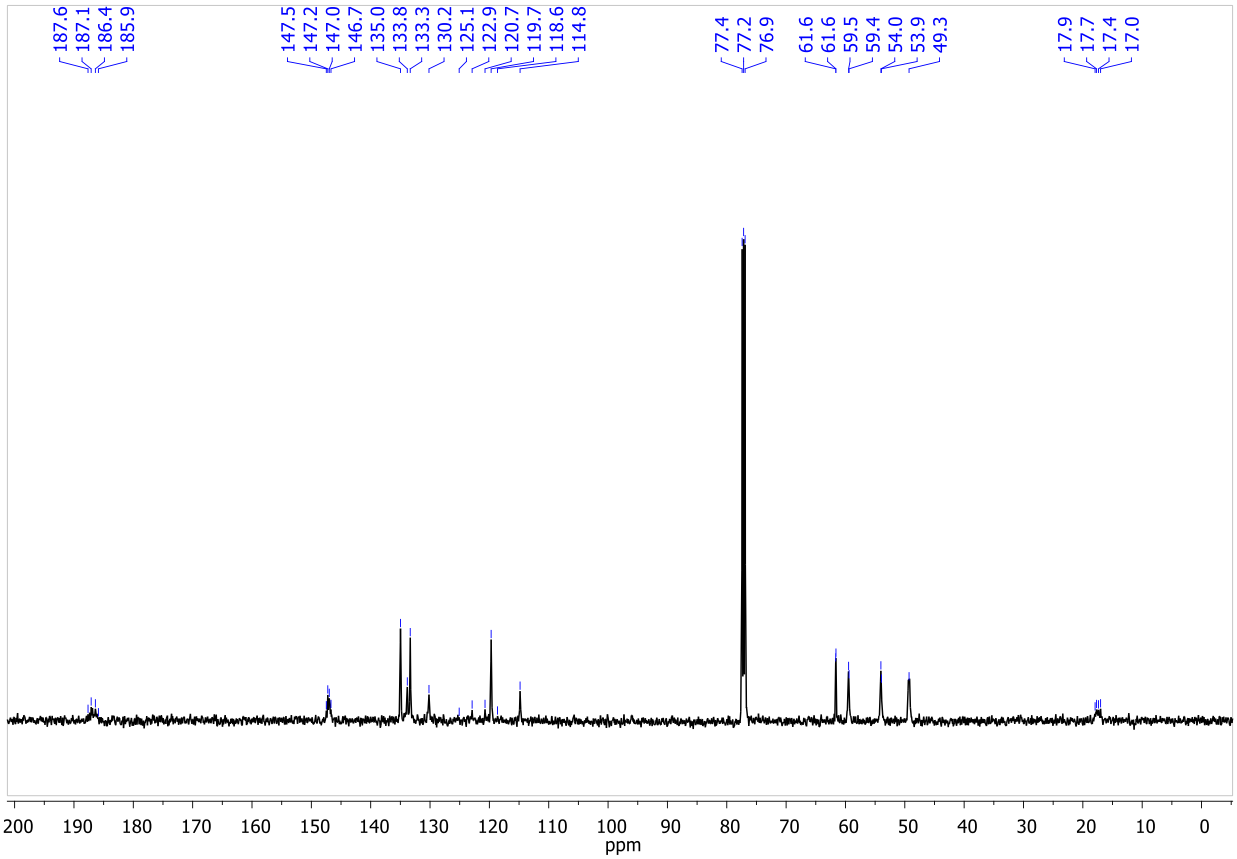

Figure S13. $^{13}C\{^1H\}$ NMR of $[MeB(6-(CF_3)Py)_3]Rh(nbd)$ (**16**) in $CDCl_3$ at -10 °C

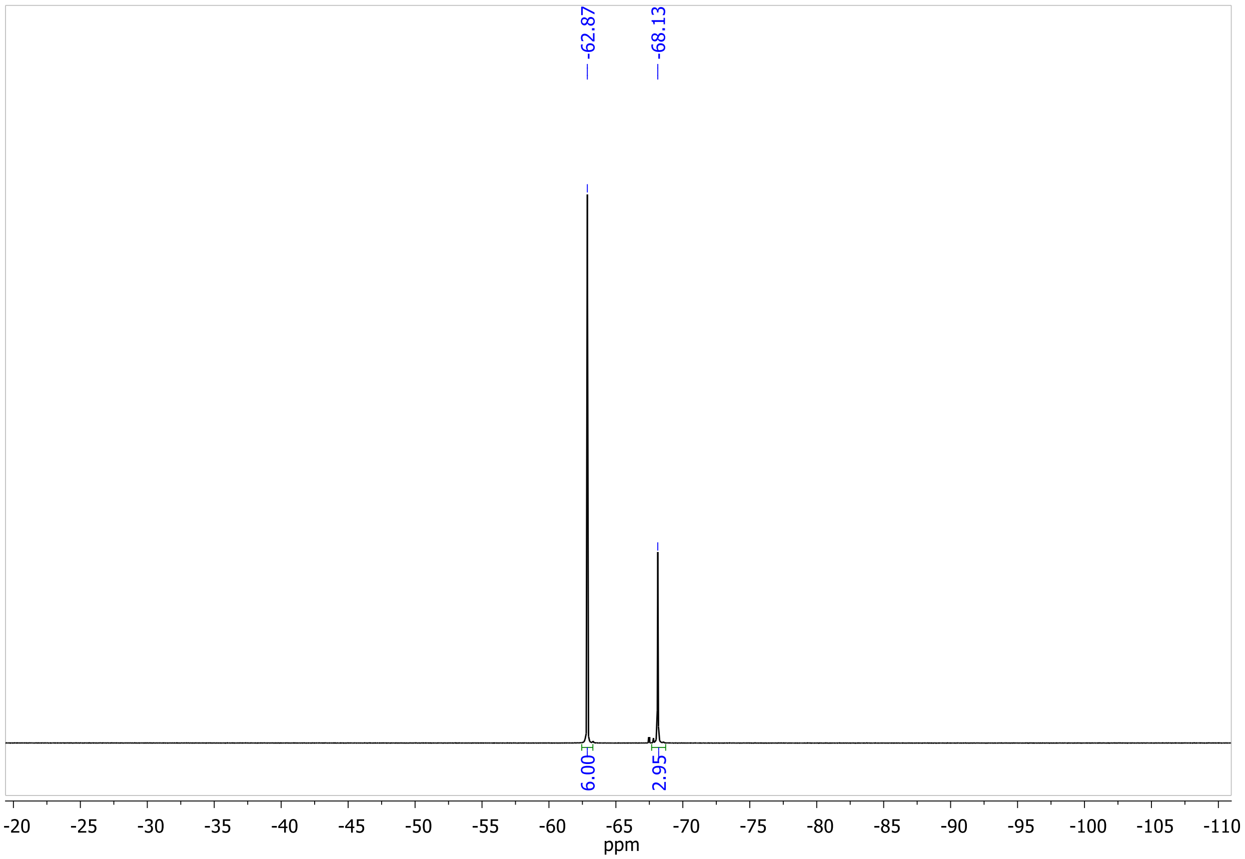

Figure S14. ^{19}F NMR of [MeB(6-(CF_3)Py)$_3$]Rh(nbd) (**16**) in $CDCl_3$ at -10 °C

Analysis of topographic steric map

Steric maps and percent buried volumes (%V_{Bur}) were computed using SambVca[1] 2.1 for a sphere radius of 3.5 Å about the metal center, Bondi van der Waals radii scaled by a factor of 1.17, 0.10 Å mesh spacing, and including hydrogen atoms (difference in %V_{Bur} value was negligible (≤0.1%) between inclusion or omission of H-atoms in these molecules during calculation). Per SambVca protocol, metal-cyclooctadiene and metal-norbornadiene moieties were removed for the calculation of steric maps and %V_{Bur} of supporting ligands in compounds **13, 14, 15, 16** and [HB(3,5-(CF_3)$_2$Pz)$_3$]Rh(nbd), illustrated in Figures S15 and S16.

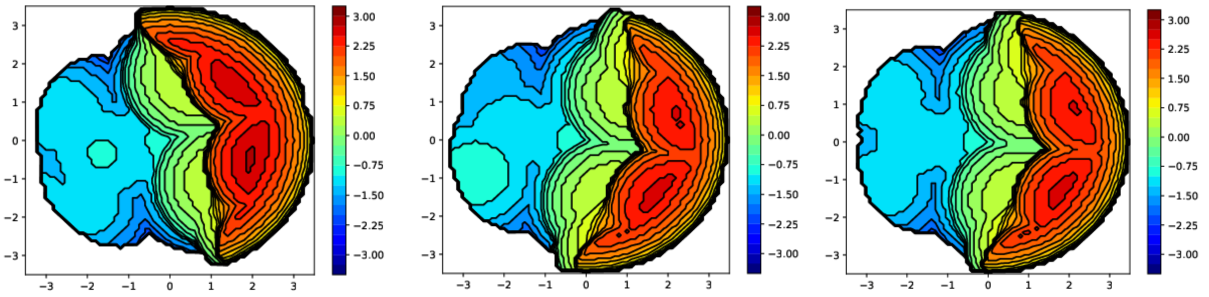

Figure S15. Steric maps of the supporting ligands in [MeB(6-(CF_3)Py)$_3$]Rh(cod) (**13**, left), in [PhB(6-(CF_3)Py)$_3$]Rh(cod) (**14**, middle), and [Ph_2B(6-(CF_3)Py)$_2$]Rh(cod) (**15**, right), looking down the Rh···B axis. The resulting % buried volume values are 53.5%, 52.9% (average for the two molecules in the asymmetric unit) and 52.7%, respectively.

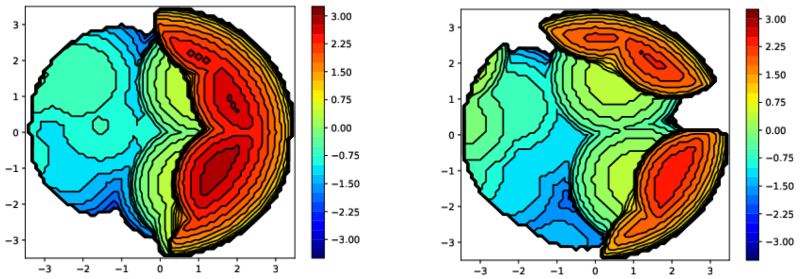

Figure S16. Steric maps of the tris(pyridyl)borate ligand in [MeB(6-(CF_3)Py)$_3$]Rh(nbd) (**16**), and tris(pyrazolyl)borate ligand in [HB(3,5-(CF_3)$_2$Pz)$_3$]Rh(nbd) supported by ligand **9**, looking down the Rh···B axis. The resulting % buried volume values are 56.4%, and 51.9%, respectively.

Analysis of topographic steric maps – nbd and cod comparison

Steric maps and percent buried volumes (%V_{Bur}) were computed using SambVca[1] 2.1 for a sphere radius of 3.5 Å about the metal center, Bondi van der Waals radii scaled by a factor of 1.17, 0.10 Å mesh spacing, and including hydrogen atoms (difference in %V_{Bur} value was negligible (≤0.1%) between inclusion or omission of H-atoms in these molecules during calculation). Per SambVca protocol, [MeB(6-(CF_3)Py)$_3$]Rh was removed for the calculation of steric maps and %V_{Bur} of cod and nbd groups in **13** and **16**.

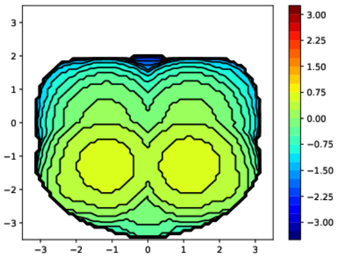

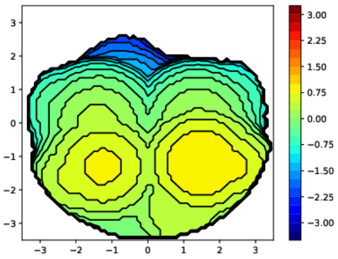

Figure S17. Steric maps of norbornadiene (nbd) and cyclooctadiene (cod) in rhodium complexes (**13** and **16**) supported by [MeB(6-(CF_3)Py)$_3$]$^-$ ligand. The resulting % buried volume values are 42.2%, and 47.2%, respectively.

X-ray crystallography

Table S1. Crystal data and structure refinement for [MeB(6-(CF_3)Py)$_3$]Rh(cod) (**13**).

Identification code	HRD116
Empirical formula	$C_{27}H_{24}BF_9N_3Rh$
Formula weight	675.21
Temperature/K	100.00
Crystal system	monoclinic
Space group	$P2_1/n$
a/Å	9.1365(4)
b/Å	14.6841(7)
c/Å	19.0402(8)
α/°	90
β/°	97.5880(10)
γ/°	90
Volume/Å^3	2532.1(2)
Z	4
ρ_{calc}g/cm^3	1.771
μ/mm^{-1}	0.765
F(000)	1352.0
Crystal size/mm^3	0.15 × 0.12 × 0.1
Radiation	Mo Kα (λ = 0.71073)
2Θ range for data collection/°	5.132 to 65.876
Index ranges	$-13 \le h \le 13$, $-22 \le k \le 22$, $-28 \le l \le 28$
Reflections collected	61604
Independent reflections	9127 [R_{int} = 0.0206, R_{sigma} = 0.0150]
Data/restraints/parameters	9127/0/372
Goodness-of-fit on F^2	1.071
Final R indexes [I>=2σ (I)]	R_1 = 0.0234, wR_2 = 0.0509
Final R indexes [all data]	R_1 = 0.0292, wR_2 = 0.0555
Largest diff. peak/hole / e Å^{-3}	0.81/-0.81

Table S2. Crystal data and structure refinement for [PhB(6-(CF_3)Py)$_3$]Rh(cod) (**14**).

Identification code	HRD154_5
Empirical formula	$C_{32}H_{26}BF_9N_3Rh$
Formula weight	737.28
Temperature/K	100(2)
Crystal system	triclinic
Space group	P-1
a/Å	12.1242(7)
b/Å	13.8125(8)
c/Å	18.8985(10)
α/°	111.039(2)
β/°	90.589(3)
γ/°	92.354(3)
Volume/Å^3	2950.3(3)
Z	4
ρ_{calc}g/cm^3	1.66
μ/mm^{-1}	0.665
F(000)	1480.0
Crystal size/mm^3	0.32 × 0.32 × 0.18
Radiation	MoKα (λ = 0.71073)
2Θ range for data collection/°	4.62 to 61.016
Index ranges	$-17 \leq h \leq 17$, $-19 \leq k \leq 18$, $0 \leq l \leq 26$
Reflections collected	16619
Independent reflections	16619 [R_{int} = 0.0305, R_{sigma} = 0.0249]
Data/restraints/parameters	16619/66/858
Goodness-of-fit on F^2	1.239
Final R indexes [I>=2σ (I)]	R_1 = 0.0469, wR_2 = 0.1662
Final R indexes [all data]	R_1 = 0.0515, wR_2 = 0.1689
Largest diff. peak/hole / e Å^{-3}	1.11/-0.94

Table S3. Crystal data and structure refinement for $[Ph_2B(6\text{-}(CF_3)Py)_2]Rh(cod)$ (**15**).

Identification code	HRD289
Empirical formula	$C_{32}H_{28}BF_6N_2Rh$
Formula weight	668.28
Temperature/K	100.00
Crystal system	triclinic
Space group	P-1
a/Å	9.5763(5)
b/Å	10.4383(5)
c/Å	13.9335(7)
α/°	89.131(2)
β/°	87.700(2)
γ/°	88.259(2)
Volume/Å^3	1390.90(12)
Z	2
ρ_{calc}g/cm^3	1.596
μ/mm^{-1}	0.680
F(000)	676.0
Crystal size/mm^3	0.18 × 0.11 × 0.04
Radiation	Mo Kα (λ = 0.71073)
2Θ range for data collection/°	5.852 to 60.958
Index ranges	$-13 \leq h \leq 13$, $-14 \leq k \leq 14$, $-19 \leq l \leq 19$
Reflections collected	22530
Independent reflections	8306 [R_{int} = 0.0210, R_{sigma} = 0.0232]
Data/restraints/parameters	8306/0/380
Goodness-of-fit on F^2	1.096
Final R indexes [I>=2σ (I)]	R_1 = 0.0312, wR_2 = 0.0751
Final R indexes [all data]	R_1 = 0.0347, wR_2 = 0.0768
Largest diff. peak/hole / e Å^{-3}	1.84/-0.92

Table S4. Crystal data and structure refinement for [MeB(6-(CF_3)Py)$_3$]Rh(nbd) (**16**).

Identification code	dia76
Empirical formula	$C_{26}H_{20}BF_9N_3Rh$
Formula weight	659.17
Temperature/K	99.98
Crystal system	monoclinic
Space group	$P2_1/c$
a/Å	18.126(2)
b/Å	8.6684(11)
c/Å	15.6182(19)
α/°	90
β/°	104.001(2)
γ/°	90
Volume/Å^3	2381.0(5)
Z	4
ρ_{calc}g/cm^3	1.839
μ/mm^{-1}	0.811
F(000)	1312.0
Crystal size/mm^3	0.331 × 0.132 × 0.11
Radiation	Mo Kα (λ = 0.71073)
2Θ range for data collection/°	4.632 to 62.016
Index ranges	$-26 \le h \le 25, -12 \le k \le 12, -22 \le l \le 22$
Reflections collected	27136
Independent reflections	7133 [R_{int} = 0.0309, R_{sigma} = 0.0312]
Data/restraints/parameters	7133/0/378
Goodness-of-fit on F^2	1.053
Final R indexes [I>=2σ (I)]	R_1 = 0.0260, wR_2 = 0.0584
Final R indexes [all data]	R_1 = 0.0319, wR_2 = 0.0608
Largest diff. peak/hole / e Å^{-3}	0.59/-0.36

C–C silylation of cyclopropyl acetates

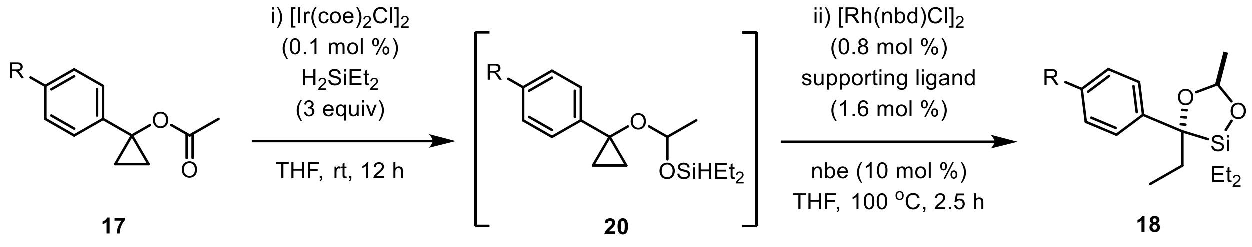

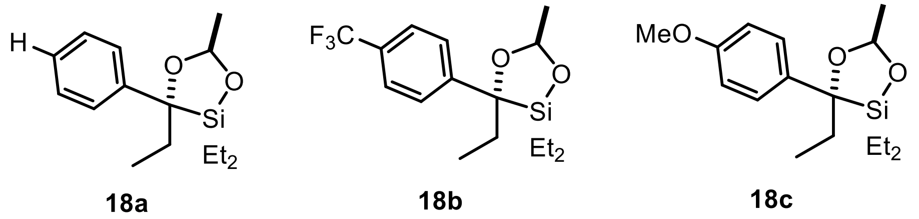

NMR data for the product 18:[2]

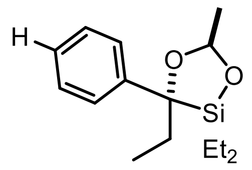

18a

^{1}H NMR (C_6D_6, 500 MHz): δ (ppm) 7.34 (d, *J* = 7.6 Hz, 2H, Ar-*H*), 7.21 (t, *J* = 7.6 Hz, 2H, Ar-*H*), 7.04 (t, *J* = 7.6 Hz, 1H, Ar-*H*), 5.32 (q, *J* = 4.8 Hz, 1H, OC*H*Me), 2.04 (dq, *J* = 14.4, 7.2 Hz, 1H, OC(Ar)CH_aH$_b$CH$_3$), 1.65 (dq, *J* = 14.4, 7.2 Hz, 1H, OC(Ar)CH$_a$$H_bCH_3$), 1.52 (d, *J* = 4.8 Hz, 3H, OCHCH_3), 1.02 (dd, *J* = 7.8, 7.8 Hz, 3H, SiCH$_2$CH_3), 0.79 (dd, *J* = 7.2, 7.2 Hz, 3H, OC(Ar)CH$_2$CH_3), 0.76 (dq, *J* = 15.7, 7.8 Hz, 1H, SiCH_aH$_b$CH$_3$), 0.66 (dd, *J* = 7.8, 7.8 Hz, 3H, SiCH$_2$CH_3), 0.63 (dq, *J* = 15.7, 7.8 Hz, 1H, SiCH$_a$$H_bCH_3$), 0.33 (dq, *J* = 15.7, 7.8 Hz, 1H, SiCH_aH$_b$CH$_3$), 0.29 (dq, *J* = 15.7, 7.8 Hz, 1H, SiCH$_a$$H_bCH_3$).

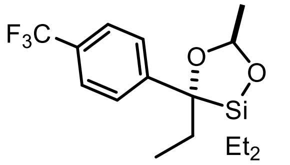

18b

^{1}H NMR (C_6D_6, 500 MHz): d 7.26 (d, *J* = 8.8 Hz, 2H, Ar-*H*), 6.85 (d, *J* = 8.8 Hz, 2H, Ar-*H*), 5.33 (q, *J* = 4.8 Hz, 1H, OC*H*Me), 3.33 [s, 3H, Ar(OMe)], 2.07 [dq, *J* = 14.6, 7.1 Hz, 1H, OC(Ar)C$\mathit{H_a}$H$_b$CH3], 1.63 [dq, *J* = 14.6, 7.3 Hz, 1H, OC(Ar)CHa$\mathit{H_b}$CH$_3$], 1.54 (d, *J* = 4.8 Hz, 3H, OCH$\mathit{CH_3}$), 1.05 (dd, *J* = 8.0, 8.0 Hz, 3H, SiCH$_2$$\mathit{CH_3}$), 0.83 [dd, *J* = 7.3, 7.3 Hz, 3H, OC(Ar)CH$_2$$\mathit{CH_3}$], 0.79 (dq, *J* = 15.9, 8.0 Hz, 1H, SiC$\mathit{H_a}$H$_b$CH$_3$), 0.72 (dd, *J* = 8.0, 8.0 Hz, 3H, SiCH$_2$$\mathit{CH_3}$), 0.67 (dq, *J* = 15.9, 8.0 Hz, 1H, SiCH$_a$$\mathit{H_b}CH_3$), 0.40 (dq, *J* = 15.3, 8.0 Hz, 1H, SiC$\mathit{H_a}$H$_b$CH$_3$), 0.40 (water in benzene-*d$_6$*, $\mathit{H_2}$O), and 0.42-0.28 (m, 15.9, 8.0 Hz, 2H, Si$\mathit{CH_2}$CH$_3$).

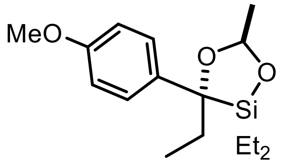

18c

^{1}H NMR (C_6D_6, 500 MHz): d 7.43 (d, *J* = 8.1 Hz, 2H, Ar-*H*), 7.20 (d, *J* = 8.1 Hz, 2H, Ar-*H*), 5.22 (q, *J* = 4.8 Hz, 1H, OC*H*Me), 1.95 [dq, *J* = 14.3, 7.1 Hz, 1H, OC(Ar)C*Ha*H$_b$CH$_3$], 1.51 [dq, *J* = 14.6, 7.1 Hz, 1H, OC(Ar)CH$_a$$\mathit{H_b}CH_3$], 1.47 (d, *J* = 4.8 Hz, 3H, OCHC$\mathit{H_3}$), 0.97 (dd, *J* = 7.9, 7.9 Hz, 3H, SiCH$_2$C$\mathit{H_3}$), 0.70 (dq, *J* = 16.2, 7.9 Hz, 1H, SiC$\mathit{H_a}$H$_b$CH$_3$), 0.67 [dd, *J* = 7.1, 7.1 Hz, 3H, OC(Ar)CH$_2$C$\mathit{H_3}$], 0.57 (dq, *J* = 16.2, 7.9 Hz, 1H, SiC$\mathit{H_2}$CH$_3$), 0.55 (dd, *J* = 7.9, 7.9 Hz, 3H, SiCH$_2$C$\mathit{H_3}$), 0.40 (water in benzene-*d*$_6$, $\mathit{H_2}$O), 0.18 (dq, *J* = 15.7, 7.9 Hz, 1H, SiC$\mathit{H_a}$H$_b$CH$_3$), and 0.11 (dq, *J* = 15.7, 7.9 Hz, 1H, SiCH$_a$$\mathit{H_b}CH_3$).

Investigation of reaction intermediates

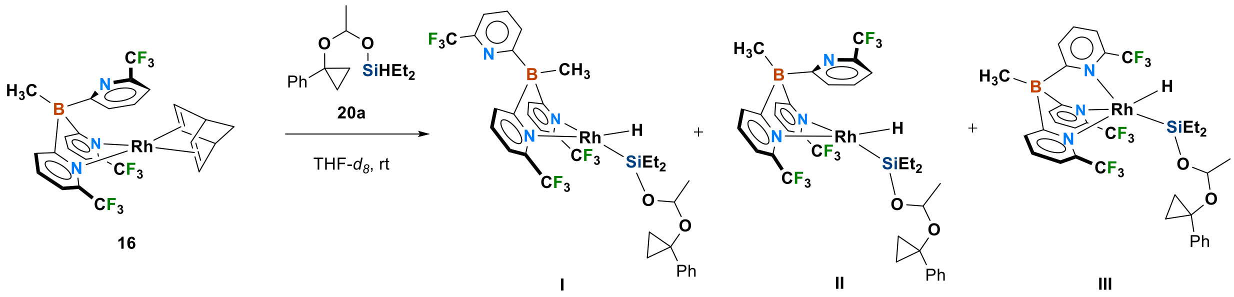

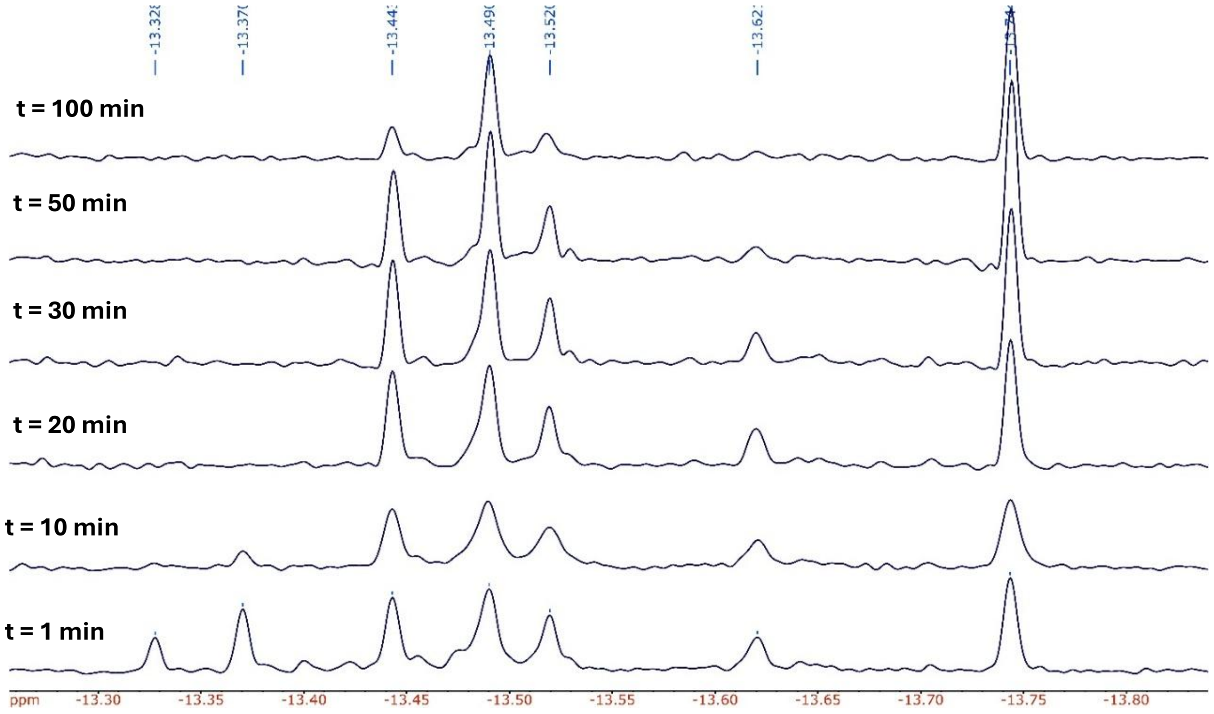

Figure S18. Likely Rh-H species that could form and ^{1}H NMR spectroscopic observations of Rh hydride species harnessing $[MeB(6\text{-}(CF_3)Py)_3]^-$ ligand in rhodium-catalyzed C–C silylation directed by silyl acetals

References

[1] L. Falivene, Z. Cao, A. Petta, L. Serra, A. Poater, R. Oliva, V. Scarano, L. Cavallo, *Nat. Chem.* **2019**, *11*, 872–879.

[2] T. Avullala, P. Asgari, Y. Hua, A. Bokka, S. G. Ridlen, K. Yum, H. V. R. Dias, J. Jeon, *ACS Catalysis* **2019**, *9*, 402–408.
