# Supplementary material for: Regio‐ and Diastereoselective C–C Silylation of Cyclopropyl Acetates Harnessing Fluorinated Poly(pyridyl)Borate Rhodium Catalysts
Source: Chemistry. 2026 Jan 24;32(14):e03217. doi: 10.1002/chem.202503217 (PMC13088001; doi:10.1002/chem.202503217)

checkCIF/PLATON report

Structure factors have been supplied for datablock(s) HRD154_5, dia76a_a, hrd116_0m_a, hrd289_a

THIS REPORT IS FOR GUIDANCE ONLY. IF USED AS PART OF A REVIEW PROCEDURE FOR PUBLICATION, IT SHOULD NOT REPLACE THE EXPERTISE OF AN EXPERIENCED CRYSTALLOGRAPHIC REFEREE.

No syntax errors found. CIF dictionary Interpreting this report

Datablock: dia76a_a

```
Bond precision:   C-C = 0.0021 A           Wavelength=0.71073

Cell:            a=18.126(2)     b=8.6684(11)        c=15.6182(19)
                 alpha=90        beta=104.001(2)     gamma=90
Temperature:     100 K

                Calculated                   Reported
Volume          2381.1(5)                    2381.0(5)
Space group     P 21/c                       P 1 21/c 1
Hall group      -P 2ybc                      -P 2ybc
Moiety formula  C26 H20 B F9 N3 Rh           C26 H20 B F9 N3 Rh
Sum formula     C26 H20 B F9 N3 Rh           C26 H20 B F9 N3 Rh
Mr              659.17                       659.17
Dx,g cm-3       1.839                        1.839
Z               4                            4
Mu (mm-1)       0.811                        0.811
F000            1312.0                       1312.0
F000'           1308.38
h,k,lmax        26,12,22                     26,12,22
Nref            7594                         7133
Tmin,Tmax       0.879,0.915                  0.685,0.746
Tmin'           0.765

Correction method= # Reported T Limits: Tmin=0.685 Tmax=0.746
AbsCorr = MULTI-SCAN
```

```
Data completeness= 0.939            Theta(max)= 31.008

R(reflections)= 0.0260( 6208)                           wR2(reflections)=
                                                        0.0608( 7133)
S = 1.053                  Npar= 378
```

The following ALERTS were generated. Each ALERT has the format
test-name_ALERT_alert-type_alert-level.
Click on the hyperlinks for more details of the test.

Alert level G

```
PLAT164_ALERT_4_G Nr. of Refined C-H H-Atoms in Heavy-Atom Struct.          4 Note
PLAT232_ALERT_2_G Hirshfeld Test Diff (M-X)  Rh       --N2       .        5.3 s.u.
PLAT910_ALERT_3_G Missing FCF Reflection(s) Below Theta(Min)[Deg]=       2.32 Note
                1  0  0,
PLAT912_ALERT_4_G Missing # of FCF Reflections Above STh/L=  0.600        460 Note
PLAT933_ALERT_2_G Number of HKL-OMIT Records in Embedded .res File          1 Note
                1  0  0,
PLAT941_ALERT_3_G Average HKL Measurement Multiplicity ..........         3.8 Low
PLAT969_ALERT_5_G The 'Henn et al.' R-Factor-gap value ..........       2.101 Note
              Predicted wR2: Based on SigI**2  2.90 or SHELX Weight  5.78
PLAT978_ALERT_2_G Number C-C Bonds with Positive Residual Density.         16 Info
```

---

0 **ALERT level A** = Most likely a serious problem - resolve or explain
0 **ALERT level B** = A potentially serious problem, consider carefully
0 **ALERT level C** = Check. Ensure it is not caused by an omission or oversight
8 **ALERT level G** = General information/check it is not something unexpected

0 ALERT type 1 CIF construction/syntax error, inconsistent or missing data
3 ALERT type 2 Indicator that the structure model may be wrong or deficient
2 ALERT type 3 Indicator that the structure quality may be low
2 ALERT type 4 Improvement, methodology, query or suggestion
1 ALERT type 5 Informative message, check

Datablock: hrd116_0m_a

Bond precision:	C-C = 0.0019 A		Wavelength=0.71073
Cell:	a=9.1365(4)	b=14.6841(7)	c=19.0402(8)
	alpha=90	beta=97.588(1)	gamma=90
Temperature:	100 K		

	Calculated	Reported
Volume	2532.1(2)	2532.1(2)
Space group	P 21/n	P 1 21/n 1
Hall group	-P 2yn	-P 2yn
Moiety formula	C27 H24 B F9 N3 Rh	C27 H24 B F9 N3 Rh
Sum formula	C27 H24 B F9 N3 Rh	C27 H24 B F9 N3 Rh
Mr	675.21	675.21
Dx,g cm-3	1.771	1.771
Z	4	4
Mu (mm-1)	0.765	0.765
F000	1352.0	1352.0
F000'	1348.39	
h,k,lmax	13,22,29	13,22,28
Nref	9492	9127
Tmin,Tmax	0.896,0.926	0.705,0.747
Tmin'	0.892	

Correction method= # Reported T Limits: Tmin=0.705 Tmax=0.747
AbsCorr = MULTI-SCAN

Data completeness= 0.962 Theta(max)= 32.938

R(reflections)= 0.0234(8271) wR2(reflections)= 0.0555(9127)

S = 1.071 Npar= 372

The following ALERTS were generated. Each ALERT has the format
test-name_ALERT_alert-type_alert-level.
Click on the hyperlinks for more details of the test.

Alert level C

```
PLAT911_ALERT_3_C Missing FCF Refl Between Thmin & STh/L=    0.600          2 Report
                 0  2  0,   1  0  1,
```

Alert level G

```
PLAT232_ALERT_2_G Hirshfeld Test Diff (M-X)  Rh       --N1       .        7.0 s.u.
PLAT232_ALERT_2_G Hirshfeld Test Diff (M-X)  Rh       --N2       .        6.0 s.u.
PLAT434_ALERT_2_G Short Inter HL..HL Contact F3       ..F6       .       2.78 Ang.
                                                -1+x,y,z  =      1_455 Check
PLAT910_ALERT_3_G Missing FCF Reflection(s) Below Theta(Min)[Deg]=       2.57 Note
                -1  0  1,   0  1  1,   0  0  2,
PLAT912_ALERT_4_G Missing # of FCF Reflections Above STh/L=  0.600        354 Note
PLAT933_ALERT_2_G Number of HKL-OMIT Records in Embedded .res File          2 Note
                 1  0  1,  -1  0  1,
PLAT969_ALERT_5_G The 'Henn et al.' R-Factor-gap value ..........       3.872 Note
              Predicted wR2: Based on SigI**2  1.43 or SHELX Weight  5.18
PLAT978_ALERT_2_G Number C-C Bonds with Positive Residual Density.         16 Info
```

0 **ALERT level A** = Most likely a serious problem - resolve or explain
0 **ALERT level B** = A potentially serious problem, consider carefully
1 **ALERT level C** = Check. Ensure it is not caused by an omission or oversight
8 **ALERT level G** = General information/check it is not something unexpected

0 ALERT type 1 CIF construction/syntax error, inconsistent or missing data
5 ALERT type 2 Indicator that the structure model may be wrong or deficient
2 ALERT type 3 Indicator that the structure quality may be low
1 ALERT type 4 Improvement, methodology, query or suggestion
1 ALERT type 5 Informative message, check

Datablock: HRD154_5

Bond precision: C-C = 0.0095 A Wavelength=0.71073

Cell:	a=12.1242(7)	b=13.8125(8)	c=18.8985(10)
	alpha=111.039(2)	beta=90.589(3)	gamma=92.354(3)
Temperature:	100 K		

	Calculated	Reported
Volume	2950.3(3)	2950.3(3)
Space group	P -1	P -1
Hall group	-P 1	-P 1
Moiety formula	C32 H26 B F9 N3 Rh	?
Sum formula	C32 H26 B F9 N3 Rh	C32 H26 B F9 N3 Rh
Mr	737.28	737.28
Dx,g cm-3	1.660	1.660
Z	4	4
Mu (mm-1)	0.665	0.665
F000	1480.0	1480.0
F000’	1476.44	
h,k,lmax	17,19,26	17,19,26
Nref	18018	16619
Tmin,Tmax	0.808,0.887	0.815,0.890
Tmin’	0.808	

Correction method= # Reported T Limits: Tmin=0.815 Tmax=0.890
AbsCorr = MULTI-SCAN

Data completeness= 0.922 Theta(max)= 30.508

R(reflections)= 0.0469(15577) wR2(reflections)= 0.1689(16619)

S = 1.239 Npar= 858

The following ALERTS were generated. Each ALERT has the format
test-name_ALERT_alert-type_alert-level.
Click on the hyperlinks for more details of the test.

Alert level C

```
PLAT029_ALERT_3_C _diffrn_measured_fraction_theta_full value Low .      0.964 Why?
PLAT220_ALERT_2_C NonSolvent   Resd 2  C   Ueq(max)/Ueq(min) Range        3.5 Ratio
PLAT241_ALERT_2_C High   'MainMol' Ueq as Compared to Neighbors of       C31A Check
PLAT342_ALERT_3_C Low Bond Precision on  C-C Bonds ...............     0.0095 Ang.
PLAT411_ALERT_2_C Short Inter H...H Contact  H30      ..H30      .       2.03 Ang.
                                                2-x,2-y,3-z  =      2_778 Check
PLAT767_ALERT_4_C INS Embedded LIST 6 Instruction Should be LIST 4     Please Check
PLAT790_ALERT_4_C Centre of Gravity not Within Unit-Cell: Resd.  #          1 Note
              C32 H26 B F9 N3 Rh
PLAT906_ALERT_3_C Large K Value in the Analysis of Variance ......      2.445 Check
PLAT910_ALERT_3_C Missing FCF Reflection(s) Below Theta(Min)[Deg]=       2.31 Note
                1  0  0,  -1  1  0,   0  1  0,   0 -1  1,   1 -1  1,  -1  0  1,
                0  0  1,   1  0  1,   0  1  1,   0 -1  2,
PLAT911_ALERT_3_C Missing FCF Refl Between Thmin & STh/L=    0.600        378 Report
                1  1  0,  10  5  0,  11  5  0, -12  6  0,  11  8  0,   7 11  0,
                8 11  0,   9 11  0,   6 12  0,   7 12  0,   8 12  0,  -6 14  0,
               -5 14  0,  -4 14  0,  -3 14  0,  -4 15  0,  -3 15  0,   5-15  1,
                3-14  1,   4-14  1,   5-14  1,   6-14  1,   7-14  1,   7-13  1,
                8-13  1,  14 -5  1, -12 -4  1, -11 -4  1, -10 -4  1,  -1 -1  1,
                   (  348 More Missing: see the .ckf listing file)
PLAT918_ALERT_3_C Reflection(s) with I(obs) much Smaller I(calc) .          1 Check
                0  0  2,
```

Alert level G

```
PLAT002_ALERT_2_G Number of Distance or Angle Restraints on AtSite          7 Note
PLAT003_ALERT_2_G Number of Uiso or U(i,j)  Restrained non-H-Atoms          6 Report
PLAT066_ALERT_1_G Predicted and Reported Tmin&Tmax Range Identical          ? Check
PLAT083_ALERT_2_G SHELXL Second Parameter in WGHT  Unusually Large      31.21 Why ?
PLAT176_ALERT_4_G The CIF-Embedded .res File Contains SADI Records          2 Report
PLAT178_ALERT_4_G The CIF-Embedded .res File Contains SIMU Records          1 Report
PLAT186_ALERT_4_G The CIF-Embedded .res File Contains ISOR Records          1 Report
PLAT187_ALERT_4_G The CIF-Embedded .res File Contains RIGU Records          1 Report
PLAT188_ALERT_3_G A Non-default SIMU Restraint Value has been used     0.0020 Report
PLAT190_ALERT_3_G A Non-default RIGU Restraint Value for First Par     0.0030 Report
PLAT190_ALERT_3_G A Non-default RIGU Restraint Value for SecondPar     0.0030 Report
PLAT191_ALERT_3_G A Non-default SADI Restraint Value has been used     0.0400 Report
PLAT242_ALERT_2_G Low    'MainMol' Ueq as Compared to Neighbors of       C18A Check
PLAT301_ALERT_3_G Main Residue  Disorder .............(Resd    1)        7% Note
PLAT860_ALERT_3_G Number of Least-Squares Restraints ............         66 Note
PLAT870_ALERT_4_G ALERTS Related to Twinning Effects Suppressed ..          ! Info
PLAT912_ALERT_4_G Missing # of FCF Reflections Above STh/L=  0.600       1545 Note
PLAT913_ALERT_3_G Missing # of Very Strong Reflections in FCF ....          2 Note
                0  1  1,   0  2  2,
PLAT933_ALERT_2_G Number of HKL-OMIT Records in Embedded .res File          5 Note
               -6  7  5,  -6  7  5,  -6 -7 14,  -6 -7 14,  -1  1  0,
PLAT941_ALERT_3_G Average HKL Measurement Multiplicity ..........         1.0 Low
PLAT969_ALERT_5_G The 'Henn et al.' R-Factor-gap value ..........      6.822 Note
              Predicted wR2: Based on SigI**2  2.48 or SHELX Weight 13.63
```

```
 0 ALERT level A = Most likely a serious problem - resolve or explain
 0 ALERT level B = A potentially serious problem, consider carefully
11 ALERT level C = Check. Ensure it is not caused by an omission or oversight
21 ALERT level G = General information/check it is not something unexpected

 1 ALERT type 1 CIF construction/syntax error, inconsistent or missing data
 8 ALERT type 2 Indicator that the structure model may be wrong or deficient
14 ALERT type 3 Indicator that the structure quality may be low
 8 ALERT type 4 Improvement, methodology, query or suggestion
 1 ALERT type 5 Informative message, check
```

Datablock: hrd289_a

Bond precision: C-C = 0.0031 A Wavelength=0.71073

Cell:	a=9.5763(5)	b=10.4383(5)	c=13.9335(7)
	alpha=89.131(2)	beta=87.700(2)	gamma=88.259(2)
Temperature:	100 K		

	Calculated	Reported
Volume	1390.90(12)	1390.90(12)
Space group	P -1	P -1
Hall group	-P 1	-P 1
Moiety formula	C32 H28 B F6 N2 Rh	C32 H28 B F6 N2 Rh
Sum formula	C32 H28 B F6 N2 Rh	C32 H28 B F6 N2 Rh
Mr	668.28	668.28
Dx,g cm-3	1.596	1.596
Z	2	2
Mu (mm-1)	0.680	0.680
F000	676.0	676.0
F000’	674.12	
h,k,lmax	13,14,19	13,14,19
Nref	8462	8306
Tmin,Tmax	0.914,0.973	0.693,0.746
Tmin’	0.885	

Correction method= # Reported T Limits: Tmin=0.693 Tmax=0.746
AbsCorr = MULTI-SCAN

Data completeness= 0.982 Theta(max)= 30.479

R(reflections)= 0.0312(7743) wR2(reflections)= 0.0768(8306)

S = 1.096 Npar= 380

```
The following ALERTS were generated. Each ALERT has the format
       test-name_ALERT_alert-type_alert-level.
Click on the hyperlinks for more details of the test.
```

Alert level C

```
PLAT031_ALERT_4_C Refined Extinction Parameter Within Range of ...      2.800 Sigma
PLAT910_ALERT_3_C Missing FCF Reflection(s) Below Theta(Min)[Deg]=       2.93 Note
                1  0  0,   0  1  0,   1  1  0,   0 -1  1,  -1  0  1,   0  0  1,
                1  0  1,   0  1  1,
PLAT911_ALERT_3_C Missing FCF Refl Between Thmin & STh/L=    0.600         12 Report
               -1-11  1,  -5-11  2,  -9  4  2,   1  1  3,   4 -1  4,  -9  3  4,
                9 -5  5,  -5 -1  9,   4  7 10,   6 -3 11,  -4  1 13,  -2  1 14,
```

Alert level G

```
PLAT154_ALERT_1_G The s.u.'s on the Cell Angles are Equal ..(Note)      0.002 Degree
PLAT434_ALERT_2_G Short Inter HL..HL Contact F2       ..F2       .       2.77 Ang.
                                                2-x,-y,2-z  =      2_757 Check
PLAT912_ALERT_4_G Missing # of FCF Reflections Above STh/L=  0.600        136 Note
PLAT933_ALERT_2_G Number of HKL-OMIT Records in Embedded .res File          3 Note
                0  1  1,   1  1  0,   4 -1  4,
PLAT941_ALERT_3_G Average HKL Measurement Multiplicity ..........         2.7 Low
PLAT969_ALERT_5_G The 'Henn et al.' R-Factor-gap value ..........       3.309 Note
              Predicted wR2: Based on SigI**2  2.32 or SHELX Weight  7.01
PLAT978_ALERT_2_G Number C-C Bonds with Positive Residual Density.         11 Info
```

```
   0 ALERT level A = Most likely a serious problem - resolve or explain
   0 ALERT level B = A potentially serious problem, consider carefully
   3 ALERT level C = Check. Ensure it is not caused by an omission or oversight
   7 ALERT level G = General information/check it is not something unexpected

   1 ALERT type 1 CIF construction/syntax error, inconsistent or missing data
   3 ALERT type 2 Indicator that the structure model may be wrong or deficient
   3 ALERT type 3 Indicator that the structure quality may be low
   2 ALERT type 4 Improvement, methodology, query or suggestion
   1 ALERT type 5 Informative message, check
```

It is advisable to attempt to resolve as many as possible of the alerts in all categories. Often the minor alerts point to easily fixed oversights, errors and omissions in your CIF or refinement strategy, so attention to these fine details can be worthwhile. It is up to the individual to critically assess their own results and, if necessary, seek expert advice.

PLATON version of 26/09/2025; check.def file version of 20/09/2025

duplicate check

No duplication found

Datablock dia76a_a - ellipsoid plot

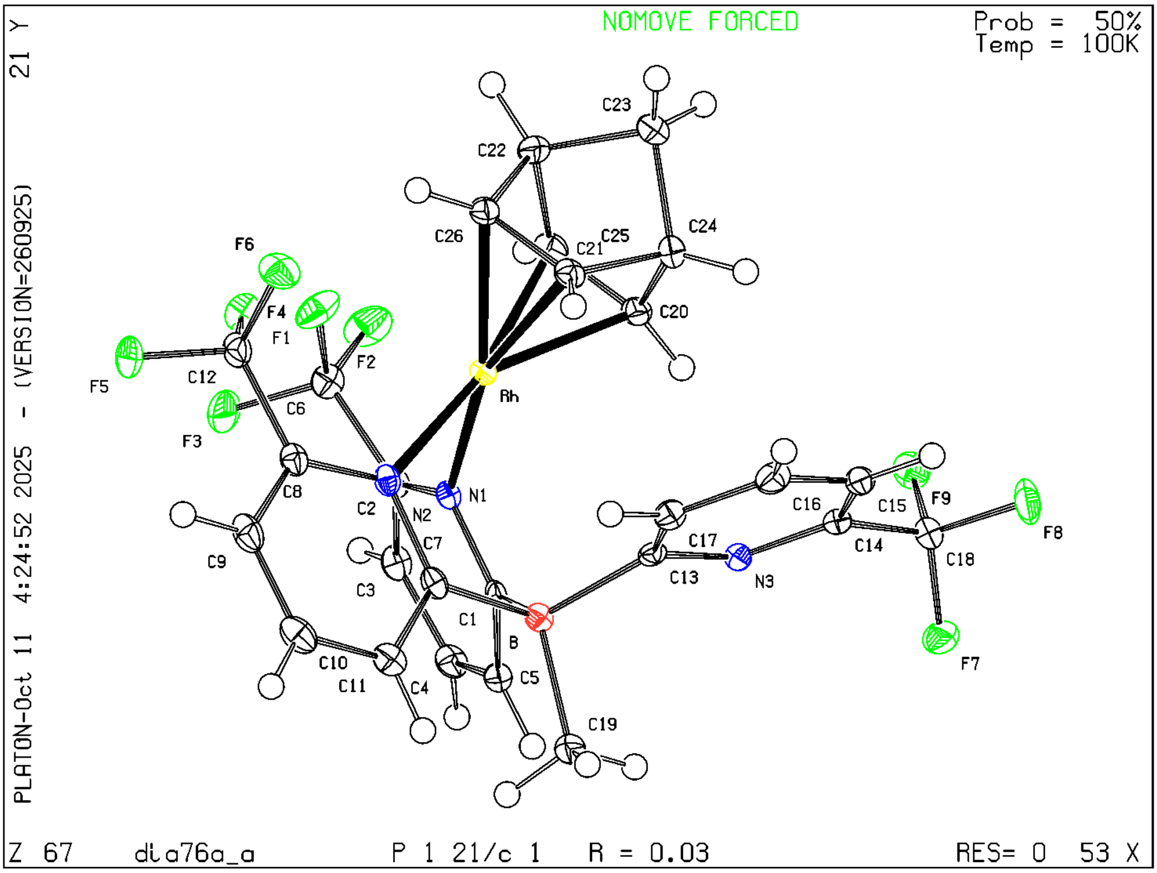

Datablock hrd116_0m_a - ellipsoid plot

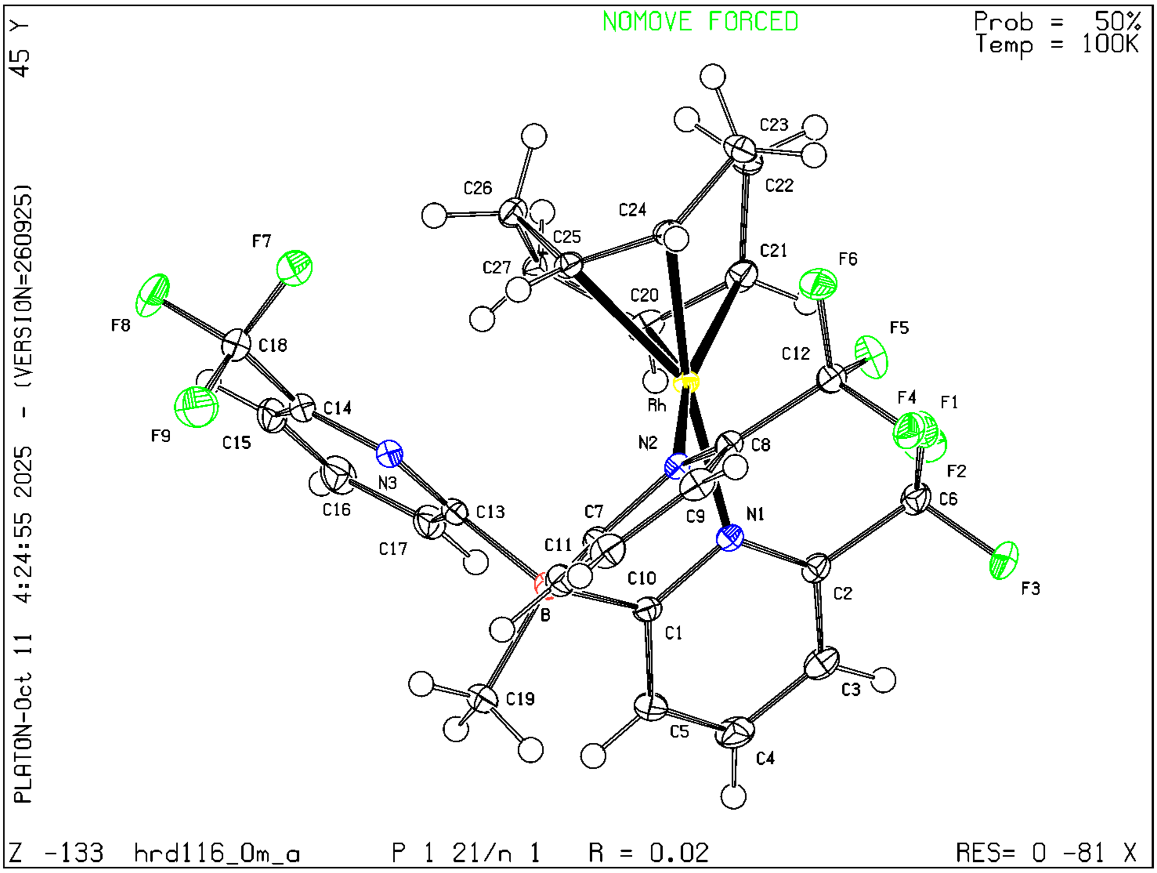

Datablock HRD154_5 - ellipsoid plot

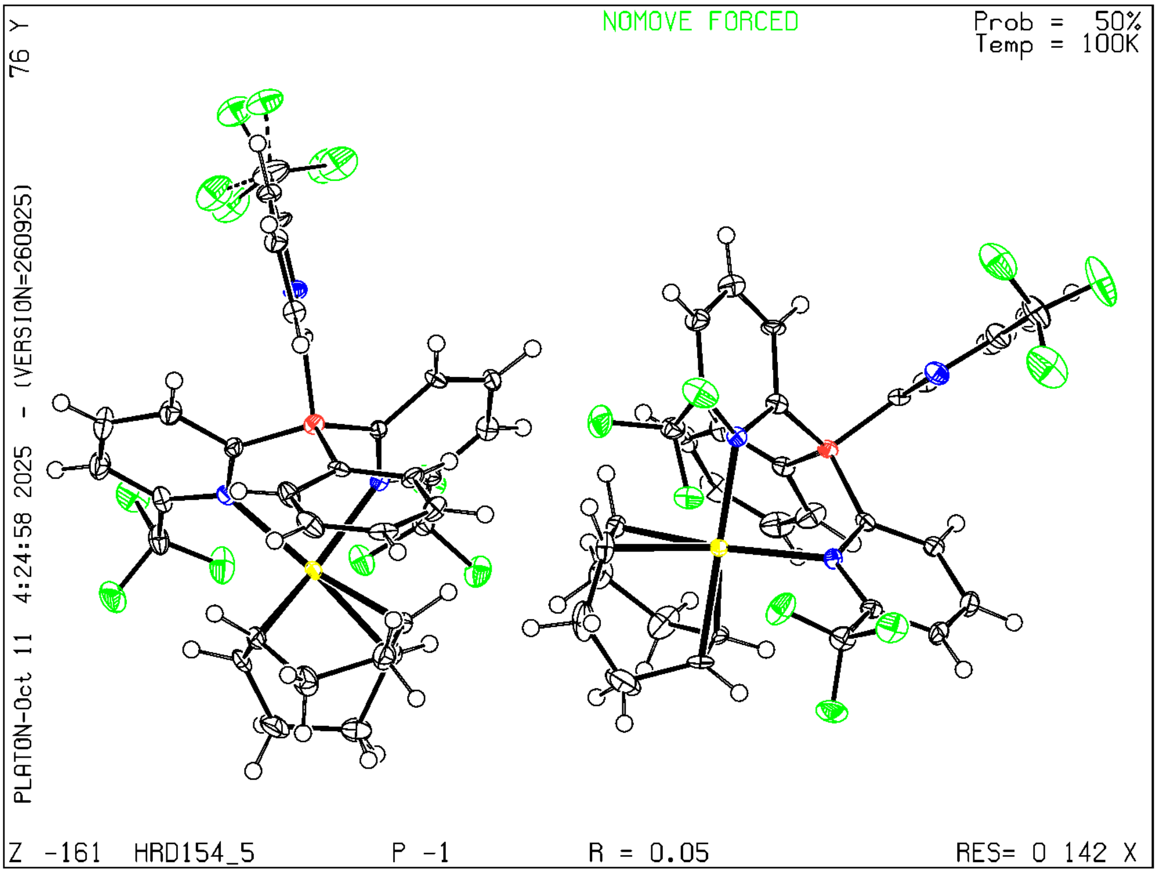

Datablock hrd289_a - ellipsoid plot

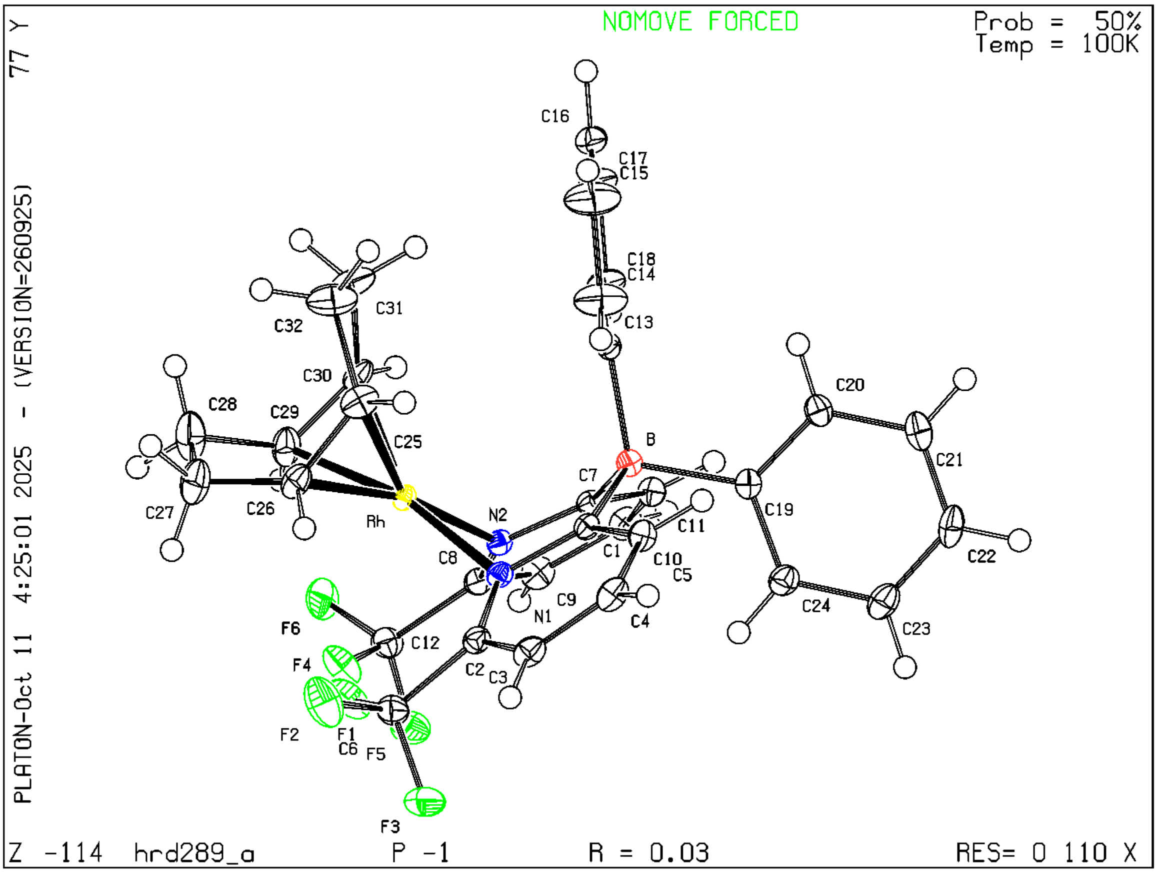

Supplement: Supplementary file 2 — Supporting File 1: chem70722‐sup‐0002‐SuppMat.pdf. [file CHEM-32-e03217-s002.pdf]
